# Diagnostic Performance of Photon-Counting CT Angiography in Vascular Stenosis Assessment: A Systematic Review and Meta-Analysis

**DOI:** 10.3390/diagnostics16060881

**Published:** 2026-03-16

**Authors:** Nasser M. Alzahrani, Awad Alzahrani, Zyad M. Almutlaq, Ahmed Alghamdi, Yazeed Almukhlifi, Sultan A. Alotaibi, Jaber Alyami

**Affiliations:** 1 Radiologic Sciences Department, Faculty of Applied Medical Sciences, King Abdulaziz University, Jeddah, Saudi Arabia; 2 Radiology and Medical Imaging Department, College of Applied Medical Sciences, King Saud Bin Abdulaziz University of Health Sciences, Riyadh P.O. Box 3660, Saudi Arabia; 3 King Abdullah International Medical Research Center, Ministry of National Guard Health Affairs, Riyadh 22384, Saudi Arabia; 4 Diagnostic Radiology Department, College of Applied Medical Sciences, King Khalid University, Abha 61421, Saudi Arabia; 5 Department of Radiological Sciences and Medical Imaging, College of Applied Medical Sciences, Majmaah University, Al Majmaah 15341, Saudi Arabia; 6 King Fahd Medical Research Center, King Abdulaziz University, Jeddah 22252, Saudi Arabia; 7 Smart Medical Imaging Research Group, King Abdulaziz University, Jeddah 22252, Saudi Arabia

**Keywords:** computed tomography, CT angiography, photon-counting detector CT, systematic review, vascular stenosis

## Abstract

**Objective**: To evaluate the performance of photon-counting detector CT (PCD-CT) angiography for the detection and quantification of vascular stenosis. **Methods**: Web of Science, PubMed, and Cochrane databases were searched from January 1980 to December 2025 to identify studies assessing PCD-CT angiography for the detection and quantification of vascular stenosis, using invasive angiography as the reference standard. The risk of bias of the included studies was assessed using the Quality Assessment of Diagnostic Accuracy Studies-2 (QUADAS-2) tool. Diagnostic performance metrics, including sensitivity and specificity and quantification values, were extracted from the included studies and a formal narrative synthesis was performed. The meta-analysis included studies reporting true-positive, false-positive, true-negative, and false-negative data. A meta-analysis was conducted only when a minimum of two eligible studies assessed diagnostic performance within the given vascular territory. Statistical analyses were performed using R software (v4.5.0), applying a random-effects model for the meta-analysis. **Results:** Of 415 identified studies, 20 were included in the systematic review, comprising a total of 9165 participants, with the majority (17/20, 85%) focusing on coronary artery stenosis. In the meta-analysis of three studies, ultra-high-resolution (UHR) PCD-CT demonstrated excellent diagnostic performance for detecting coronary stenosis for patients with ≥50%, having a pooled sensitivity of 96.1% (95% confidence level (CI): 89.3–99.6), specificity of 87.5% (95% CI: 78.2–93.3), positive predictive value (PPV) of 91.9% (95% CI: 70.3–98.2), and negative predictive value (NPV) of 94.8% (95% CI: 86.0–98.6). Compared with conventional energy-integrating detector CT (EID-CT), PCD-CT consistently showed superior diagnostic performance, particularly in the specificity and PPV. In terms of stenosis quantification, PCD-CT showed closer agreement with reference standards than EID-CT, leading to the reclassification of coronary stenosis severity in up to 49% of patients. Evidence for non-coronary vascular territories, including intracranial and peripheral arteries remains limited but suggests promising diagnostic performance. For lower-limb arterial stenosis, the reported sensitivity was 77.4–91%, and specificity was 72.1–91%. For intracranial in-stent stenosis, PCD-CT demonstrated a sensitivity of 100% and a specificity of 89%. **Conclusions:** PCD-CT angiography provides high diagnostic performance and improved stenosis quantification for coronary artery stenosis. UHR PCD-CT has excellent diagnostic performance for detecting coronary stenosis and consistently outperforms conventional EID-CT, especially in the specificity and positive predictive value.

## 1. Introduction

Cardiovascular diseases remain the leading cause of morbidity and mortality globally. Vascular stenosis is one of the most commonly encountered vascular diseases [1]. Vascular stenosis is characterized by narrowing of the arterial lumen, primarily due to atherosclerotic plaque formation or post-interventional changes such as in-stent restenosis (ISR) [2]. The resulting luminal narrowing compromises blood flow, potentially leading to life-threatening events, including strokes, heart attacks, or limb ischemia [2]. Therefore, the early and accurate detection of stenosis is pivotal for timely clinical decision-making and appropriate intervention.

Computed tomography angiography (CTA) has become a cornerstone in the non-invasive assessment of vascular diseases [3,4]. Its non-invasive nature, widespread availability, rapid acquisition, and high diagnostic accuracy have led to its widespread use as the initial imaging tool for evaluating vascular stenosis in clinical practice across various settings [3,4]. Coronary CT angiography (CCTA), in particular, is now recommended as the first-line imaging method for patients with a low-to-intermediate risk of coronary artery disease (CAD) due to its high sensitivity and negative predictive value (NPV) [5,6]. CTA is also commonly used for treatment planning before transcatheter aortic valve replacement (TAVR) [7,8] and for evaluating coronary stent patency [9,10]. Additionally, CTA plays a valuable role in the non-invasive evaluation of peripheral arterial disease [11].

However, the diagnostic performance of conventional energy-integrating detector computed tomography (EID-CT) is compromised by several inherent limitations [12]. Excessive vascular calcifications often cause calcium-blooming artifacts, reducing the test’s positive predictive value (PPV) [11,12,13,14,15,16]. Additionally, EID-CT has a limited spatial resolution for resolving small vascular structures [17]. In the evaluation of stent patency, technical issues—including metallic, blooming, and beam-hardening artifacts—lower the accuracy of CTA [18,19], particularly in stents smaller than 3 mm in diameter [20]. Furthermore, in high-risk populations—such as those undergoing TAVR—vascular calcification and contraindications to heart-rate-lowering agents (e.g., beta-blockers) often compromise CT image quality, which is essential for a precise diagnosis [21,22]. Beyond detection, the quantification of stenosis severity on EID-CT angiography is also affected by blooming artifacts and a limited spatial resolution, as they can lead to the overestimation of stenosis [11,12,13,14], thereby influencing decisions regarding patient care and management [23]. As a result, the diagnostic utility of EID-CT angiography in patients with severe vascular calcifications or implanted stents remains limited.

Photon-counting detector computed tomography (PCD-CT) is an emerging advance in CT technology, recently introduced into clinical practice following FDA approval in 2021 for the first commercial PCD-CT scanner (NAEOTOM Alpha, Siemens Healthineers) [12,24]. Unlike conventional EID-CT, which relies on an indirect conversion process in which X-ray photons are first transformed into visible light by scintillators and then detected by a photodiode to generate an electronic signal [12,25,26], PCD-CT employs semiconductors that directly convert each X-ray photon into an electrical signal [12,25,26]. This direct conversion of photons enables counting each photon and distinguishing its energy level while effectively eliminating electronic noise [12,25,26]. As a result, PCD-CT enhances material differentiation and reduces image noise [25,26]. Furthermore, the absence of septa within the PCD-CT allows smaller pixel sizes, resulting in a higher spatial resolution [26,27]. PCD-CT offers distinct advantages that improve the diagnostic performance of CTA in evaluating stenotic lesions, particularly in high-risk patients [28]. The superior spatial resolution of PCD-CT provides the clearer visualization of small vessels and stenotic lesions [29]. Blooming artifacts from calcified plaques and beam-hardening artifacts from metallic stents are significantly reduced [24,29], allowing clearer separation between the intraluminal vessel and surrounding high-density structures [24] and thereby yielding the more accurate detection and quantification of stenotic lesions [30].

To the best of the authors’ knowledge, no systematic review has comprehensively evaluated the diagnostic performance of PCD-CT angiography for the detection or quantification of vascular stenosis. Therefore, this study aims to systematically review the current evidence on (i) the diagnostic performance of PCD-CT angiography for stenosis detection and (ii) the quantification of stenosis via PCD-CT, using invasive angiography as the reference standard.

## 2. Materials and Methods

This systematic review was conducted in accordance with the Preferred Reporting Items for Systematic Reviews and Meta-Analyses (PRISMA) statement [31]. The study protocol for this systematic review has been registered on PROSPERO under the reference number CRD420251084027.

### 2.1. Literature Search

Web of Science, PubMed, and Cochrane databases were searched for eligible articles published between January 1980 and December 2025. The search was limited to studies published in English. The detailed search strategies are provided in Supplementary Material Table S1. Reference lists of all eligible studies were also searched.

### 2.2. Study Selection

The inclusion criteria were as follows: (1) patients with suspected or known vascular stenosis (either native or in-stent stenosis); (2) use of PCD-CT angiography as a diagnostic tool to detect and/or quantify vascular stenosis; (3) use of invasive angiography as the reference standard, with stenosis quantification based on quantitative angiographic measurements; (4) reports of diagnostic accuracy metrics (e.g., sensitivity and specificity) and/or quantitative measures of stenosis (e.g., percentage diameter stenosis or level of agreement); (5) for a meta-analysis of diagnostic accuracy, the absolute numbers of true positives (TPs), true negatives (TNs), false positives (FPs), and false negatives (FNs) were reported or could be derived to calculate sensitivity and specificity. The exclusion criteria were as follows: (1) studies performed on animals and/or phantoms; (2) case reports, review articles, editorial letters, and abstracts with insufficient data to meet the inclusion criteria; (3) non-English studies. Study titles and abstracts were screened by one reviewer (N.M.A.). The full-text screening of potentially eligible studies was then independently performed by two reviewers (N.M.A. and A.S.A.), both senior CT technologists with 12 years of clinical experience and 8 years of research experience in radiology. Any discrepancies were resolved through discussion and consensus between the two reviewers (N.M.A. and A.S.A); this was required for one study during the full-text screening stage.

### 2.3. Data Extraction and Quality Assessment

Data were independently extracted from the eligible studies by three reviewers (A.A., Y.A., and S.A.) through designed data extraction and subsequently reviewed by one reviewer (N.M.A.). Any discrepancies were resolved through discussion and consensus among the four reviewers. The extracted variables included the following: author, study design, sample size, mean age, vascular territory, vessel status (i.e., native or in-stent), stenosis detection threshold, reference standard, CT imaging protocol, mean effective radiation dose, and the study’s primary outcome.

The quality of the included studies was independently assessed by three reviewers (N.M.A., A.S.A, Z.M.A.) using the Quality Assessment of Diagnostic Accuracy Studies 2 (QUADAS-2) tool [32], which evaluates four domains: patient selection, index test, reference standard, and flow and timing. Discrepancies between reviewers were resolved through discussion and consensus. For this systematic review, the acceptable time intervals between the index test (i.e., PCD-CT angiography) and the reference standards were ≤30 days.

### 2.4. Data Synthesis and Analysis

The narrative synthesis of the findings from the included studies was preformed, summarizing study characteristics and main outcomes regarding the detection and quantification performance of PCD-CT angiography in the evaluation of vascular stenosis, with the results presented in tables. To perform a meta-analysis, the absolute numbers of TPs, TNs, FPs, and FNs were extracted or derived from the included studies to assess the diagnostic performance of PCD-CT angiography at different analysis levels (i.e., patient-, vessel- and segment-level). When these data were not reported or could not be derived from the published articles, the corresponding study investigators were contacted to request the relevant information. A meta-analysis requires a minimum of two eligible studies within a given vascular territory. Meta-analyses were conducted for each vascular territory (coronary, lower limb, and intracranial arteries), stratified by the vessel status (native vs. in-stent), stenosis threshold (≥50% or ≥70%), and spatial resolution mode (standard, high, or ultra-high). A univariate random-effects model was utilized to produce forest plots estimating the sensitivity and specificity with 95% confidence intervals (CIs). The area under the curve (AUC) was derived from Summary Receiver Operating Characteristic (SROC) curves. Potential heterogeneity among the included studies in the meta-analysis was evaluated using the I2 statistic with values of 0–40% indicating low, 50–75% indicating moderate, and >75% indicating high heterogeneity [33]. A formal assessment of reporting bias was not performed due to the limited number of studies included in the meta-analysis. A *p*-value of less than 0.05 was deemed statistically significant. Data analysis was performed using metafor 4.8.0 and mada 0.5.12, R v4.5.0 packages.

## 3. Results

### 3.1. Search Strategy

**Figure 1:**
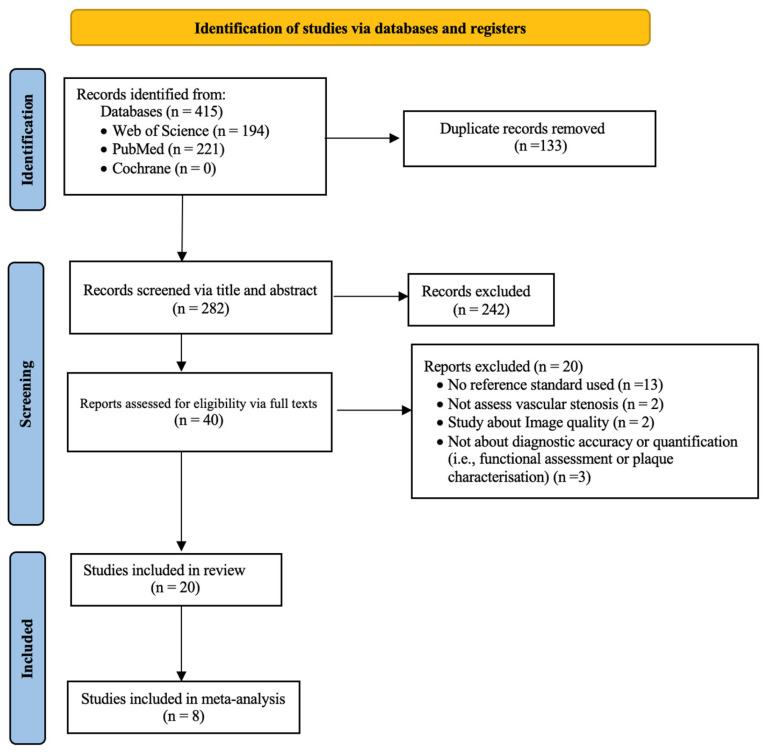
PRISMA flow diagram of study screening and selection.

The results of the literature search and the reasons for study exclusion are shown in Figure 1. A total of 415 articles were initially identified through the database searches. After removing 133 duplicates, 282 records remained for screening. Of these, 242 were excluded based on a title and abstract review, leaving 40 articles for full-text assessment. Following full-text review, 20 studies met the inclusion criteria and were included in the qualitative synthesis. Reasons for exclusion are summarized in Figure 1, with details of excluded studies (author and title) provided in Table S2 in the Supplementary Material. For a quantitative meta-analysis assessing the diagnostic performance of PCD-CT in detecting vascular stenosis, 12 of the 20 studies were excluded. Eight studies [28,29,30,34,35,36,37,38] did not report or allow derivation of TP, FP, TN, and FN values. Three studies [39,40,41] focused solely on stenosis quantification rather than detection. Only one study [42] evaluated intracranial artery stenosis and, therefore, did not meet the minimum requirement for meta-analysis.

### 3.2. Study Characteristics and Quality Assessment

Table 1 summarizes the characteristics and main findings of the included qualitative studies. The number of patients in the included studies ranged from 17 to 3876, with a total of 5182 patients. The mean age per study ranged from 58 to 83 years, with a mean age of 70.3 years across all studies. Among the 20 included studies, 14 [29,34,35,37,38,42,43,44,45,46,47,48,49,50] focused on detecting stenosis, 3 [39,40,41] focused on stenosis quantification, and 3 [28,30,36] evaluated both the detection and quantification of stenosis. Regarding the site of stenosis evaluation via PCD-CT angiography, 17 studies [28,30,35,36,37,38,39,40,41,43,44,45,46,47,48,49,50] were on coronary territories, one study [42] was on intracranial vessels, and two studies [29,34] were on lower limb arteries. Two studies [42,45] evaluated the performance of PCD-CT angiography in detecting in-stent stenosis. The majority of the included studies (19/20, 95.0%) utilized a PCD-CT scanner from Siemens Healthineers (NAEOTOM Alpha), but one study [28] employed a PCD-CT scanner from Philips Healthcare (not yet FDA approved). The mean effective radiation dose (mSv), ranging from 1.4 mSv to 20.5 mSv, for PCD-CT angiography was reported in ten studies [30,36,37,40,41,44,46,47,48,49], all of which evaluated coronary stenosis.

Table 2 summarizes the results of the quality assessment of the 20 included studies. Regarding the risk of bias in patient selection, one study [30] was scored as an unclear risk due to poor reporting of the sampling procedure. In the index test domain, three studies [38,40,41] did not provide information on whether PCD-CT results were interpreted without knowledge of reference standard results and were scored as having an unclear risk of bias. Four studies [43,44,47,50] were scored as having an unclear risk of bias in the reference standard domain because it was unclear whether the reference standard was interpreted without knowledge of the index test results. Considering flow and timing, four studies [29,35,41,42] were judged to have an unclear risk of bias because they did not report the interval between the invasive angiographic procedure (i.e., reference standard) and PCD-CT angiography. Among the ten studies assessed as having a high risk of bias, eight [37,38,39,40,43,45,49,50] exceeded the predefined acceptable interval between PCD-CT angiography and the reference standard (≤30 days). The remaining two studies [37,47] did not apply the reference standard to all enrolled patients, introducing a potential partial verification bias.

### 3.3. Quantification of Stenosis

Six studies [28,30,36,39,40,41] evaluated PCD-CT angiography in quantitative coronary stenosis measurements against quantitative coronary angiography (QCA). Two of these studies [28,40] compared PCD-CT with conventional EID-CT using QCA as the reference standard. Vecsey-Nagy et al. [40] reported that UHR PCD-CT yielded a significantly lower percent diameter stenosis than EID-CT for both calcified (45.1 ± 20.7% vs. 54.6 ± 19.2%; *p* < 0.001) and partially calcified plaques (44.3 ± 19.6% vs. 54.9 ± 20.0%; *p* < 0.001). Agreement with QCA was stronger for PCD-CT than for EID-CT (ICC = 0.91 vs. 0.83). Moreover, 49% (24/49) of patients were reclassified to a lower CAD-RADS category when assessed with PCD-CT. Fahrni et al. [28] similarly showed that agreement with QCA was markedly stronger for PCD-CT than for EID-CT (ICC = 0.94 vs. 0.65), with 38% of stenoses reclassified to correct CAD-RADS categories that had been misclassified via EID-CT.

Two studies [30,36] evaluated the effect of the spatial resolution of PCD-CT on the quantification of coronary stenosis. Laux et al. [36] demonstrated that increasing the spatial resolution from a standard resolution (SR) to UHR progressively reduced the median percent diameter of stenosis from 61.4% at SR to 55.3% at high resolution (HR) and 50.9% at UHR, approaching the QCA reference of 46.4%. Correspondingly, the mean difference between PCD-CT and QCA decreased from 13.2% with SR to 5.2% with UHR acquisition. Similarly, Kotronias, et al. [30] found that UHR PCD-CT yielded more accurate stenosis measurements than HR (median difference 3% vs. 6%; *p* < 0.001) when compared with QCA and showed a substantially lower vessel-level CAD-RADS misclassification rate (7.6% vs. 27.3%).

Two studies [39,41] evaluated the effect of photon energy and reconstruction algorithms on stenosis quantification using PCD-CT. Mergen et al. [39] compared conventional virtual monoenergetic imaging (VMI) and virtual non-calcium (VNCa) reconstruction (PureLumen algorithm) against 3D-QCA for 71 calcified stenoses. VMI consistently overestimated stenosis compared with QCA (mean difference: −10.0% and −7.0% for readers 1 and 2, respectively), while VNC images closely matched QCA (mean difference: 0–1.0% for readers 1 and 2, respectively). Wolf et al. [41] investigated multiple monoenergetic levels (40–140 keV) in 64 stenoses and demonstrated that the stenosis diameter in calcified and mixed plaques decreased with an increasing energy, whereas it increased for non-calcified plaques. The lowest mean difference for the stenosis diameter for calcified plaque was 100 keV (17.2%), for mixed plaques, it was 140 keV (5.0%), and for non-calcified plaques, it was 40 keV (−0.5%).

### 3.4. Detection of Stenosis

Supplementary Material Table S3 presents the reported diagnostic performance of PCD-CT angiography for stenosis detection in the individual studies. Using invasive angiography as the reference standard, 17 studies evaluated the diagnostic performance of PCD-CT angiography for detecting vascular stenosis, encompassing coronary (*n* = 14, [28,30,35,36,37,38,39,40,41,43,44,45,46,47,48,49,50]), peripheral (*n* = 2, [29,34]), and intracranial (*n* = 1, [42]) vascular territories.

#### 3.4.1. Coronary Arteries

For the ≥50% stenosis threshold, the diagnostic performance of PCD-CT angiography based on a patient-based analysis (772 patients) in the included studies showed sensitivity ranging from 78.0% to 100.0%, specificity from 29.6% to 95.2%, PPV from 45.0% to 97.6%, and NPV from 56.9% to 100.0%. Based on a vessel-based analysis (3249 vessels), sensitivity ranged from 67.0% to 100.0%, specificity ranged from 63.0% to 100%, PPV ranged from 35.0% to 93.1%, and NPV ranged from 89.0% to 100.0%. On a segments-based analysis (5364 segments), sensitivity ranged from 77.0% to 100.0%, specificity ranged from 69.0% to 98.7%, PPV ranged from 15.5% to 93%, and NPV ranged from 94.3% to 100.0%. 

For the ≥70% stenosis threshold, the patient-based analysis (321 patients) reported sensitivity ranging from 87.5% to 100.0%, specificity from 61.0% to 95.8%, PPV from 30.4% to 97.2%, and NPV from 92.0% to 100.0%. Vessel-based analysis (1216 vessels) showed sensitivity ranging from 75.0% to 100.0%, specificity from 85.6% to 96.5%, PPV from 18.2% to 88.9%, and NPV from 98.1% to 100%. Segments-based analysis (4270 segments) demonstrated sensitivity ranging from 63.6% to 96.2%, specificity from 84.2% to 100%, PPV from 4.9% to 100%, and NPV from 90.0% to 100%.

Three studies [28,38,46] directly compared the diagnostic performance of PCD-CT and conventional EID-CT using invasive coronary angiography (ICA) as the reference standard. PCD-CT consistently demonstrated superior diagnostic performance than EID-CT across patient-, vessel-, and segment-level analyses, particularly in specificity and PPV. The detailed comparison is presented in Supplementary Material Table S4.

Three studies [37,44,45] assessed the diagnostic performance of PCD-CT angiography for detecting ISR using ICA as the reference standard. Hagar et al. [44] reported that PCD-CT had a sensitivity of 100% and specificity of 86.0% for detecting ISR in 15 patients with prior stents. Another study by Hagar et al. [45], of 18 patients with 44 stents, reported that PCD-CT had sensitivity as 100% and specificity as 89.7%. Additionally, Qin et al. [37] reported that UHR-Bv72 PCD-CT had higher sensitivity (75.0% vs. 25%), specificity (90.0% vs. 89%), and diagnostic accuracy (88.0% vs. 78.3%) than SR PCD-CT for detecting ISR.

A total of eight studies ([43,44,45,46,47,48,49,50]) were included in the meta-analysis, all of which evaluated the diagnostic performance of PCD-CT for detecting stenosis in coronary arteries. Table 3 and Supplementary Material Figures S1–S9 show the pooled sensitivity, specificity, PPV, and NPV of PCD-CT for assessing both native and in-stent coronary stenosis across different spatial resolution modes. Both HR and UHR modes demonstrated high sensitivity and NPV across analysis levels; however, UHR outperformed HR in specificity (87.5% vs. 68.6% at patient level; 93.6% vs. 93.3% at vessel level; 97.5% vs. 87.1% at segment level) and PPV (91.9% vs. 73.8% at patient level; 85.1% vs. 74.6% at vessel level; 76.3% vs. 58.3% at segment level, respectively) (Table 3). The pooled sensitivity, specificity, PPV, and NPV of UHR PCD-CT in assessing the presence of in-stent coronary stenosis are 62.6%, 62.4%, 68.6%, and 57.2%, respectively (Table 3). Heterogeneity among studies included in the meta-analysis was low to moderate for sensitivity (I^2^ = 0–45%) and ranged from low to high for specificity (I^2^ = 0–92%).

#### 3.4.2. Peripheral Arteries

Two studies [29,34] evaluated the diagnostic accuracy of PCD-CT angiography in detecting lower limb arterial stenosis (≥60%) using a segment-based analysis across 1680 segments. Sensitivity ranged from 77.4% to 91.0%, specificity ranged from 72.1% to 91%, PPV ranged from 83.0% to 91.0%, and NPV ranged from 90.0% to 94.0%.

#### 3.4.3. Intracranial Arteries

De Beukelaer et al. [42] evaluated the diagnostic accuracy of PCD-CT angiography for detecting intracranial arterial stenosis (≥ 50%) in 162 segments. Sensitivity, specificity, PPV, and NPV were 100%, 89.0%, 53.0%, and 100%, respectively.

## 4. Discussion

PCD-CT is an emerging imaging technology with the potential to improve image quality and diagnostic confidence in assessing vascular stenoses, especially in the presence of severe calcifications or a stent. Since the first-in-human study published by Si-Mohamed et al. [51], several investigations have demonstrated the promising clinical role of PCD-CT across various applications. However, to date, no systematic reviews have been published to comprehensively evaluate its diagnostic performance. This systematic review and meta-analysis provides an overview of the diagnostic and quantification performance of PCD-CT angiography in vascular stenosis using invasive angiography as a reference standard. The vast majority of included studies in this systematic review focused on coronary artery stenosis (17 of 20 studies, 85%), reflecting the predominant clinical and research interest in evaluating the diagnostic performance of PCD-CT angiography for CAD. This strong emphasis on coronary applications underscores the potential of PCD-CT to overcome key limitations of conventional EID-CT, particularly in assessing luminal narrowing in the presence of stent or heavy calcification. With regard to stenosis quantification, PCD-CT yielded measurements that more closely approximated the reference standard, resulting in the reclassification of coronary stenosis severity in 49.0% of patients [40] and 38.0% of stenoses [28]. Furthermore, this study demonstrates the high diagnostic performance of UHR PCD-CT for detecting coronary artery stenosis in patients with ≥50% stenosis with pooled sensitivity, specificity, PPV, and NPV of 96.1% (95% CI: 89.3–99.6), 87.1% (95% CI: 78.2–93.2), 91.9% (95% CI: 70.3–98.2), and 94.8% (95% CI: 86.0–98.2), respectively. Three studies in this systematic review [28,38,46] directly compared PCD-CT with conventional EID-CT for the evaluation of coronary artery stenosis. Across these studies, PCD-CT consistently demonstrated superior diagnostic performance to EID-CT, particularly in terms of specificity and PPV. Additionally, the diagnostic performance of PCD-CT angiography for detecting peripheral arterial stenosis showed a sensitivity of 77.4–91%, specificity of 72.1–91.0%, PPV of 83.0–91.0%, and NPV of 90.0–94.0%, based on the segment-level analysis.

CTA has been used generally as the initial non-invasive imaging technique for evaluating vascular stenosis. Several studies have shown that coronary CTA-based EID-CT serves as an initial imaging tool and gatekeeper for ICA due to its high sensitivity for ruling out significant CAD; however, its specificity and PPV are relatively low and vary across studies [52,53,54,55]. A meta-analysis [54] showed that coronary CTA-based EID-CT yielded higher diagnostic performance than single-photon-emission computed tomography (SPECT) or exercise electrocardiography (ECG), using ICA with ≥50% luminal stenosis as the reference standard; coronary CTA had a sensitivity of 98.0% (95% CI: 95.0–99.0), specificity of 82.0% (95% CI: 68.0–93.0), PPV of 85.0% (95% CI: 75.0–93.0), and NPV of 97.0% (95% CI: 96.0–990). Another meta-analysis, by Knuuti et al. [52] reported that coronary CTA-based EID-CT had a sensitivity of 97.0% (95% CI: 93.0–99.0) and specificity of 78.0% (95% CI: 67.0–86.0) when compared to ICA with ≥50% luminal stenosis as the reference standard. The relatively low or variable specificity and PPV of coronary CTA performed with EID-CT are likely attributable to blooming artifacts, particularly in the presence of extensive calcification or coronary stents [46,53,56]. These artifacts can lead to overestimation of stenosis severity and, consequently, an increased false-positive rate [46,53,56]. In our meta-analysis, CCTA based on UHR PCD-CT demonstrated sensitivity, specificity, PPV, and NPV of 96.1% (95% CI: 89.3–99.6), 87.5% (95% CI: 78.2–93.2), 91.9% (95% CI: 70.3–98.2) and 94.8% (95% CI: 86.0–98.2), respectively. The improved spatial resolution of PCD-CT contributes to more accurate detection of coronary stenoses.

Blooming artifacts caused by the stent itself and the surrounding calcifications visualization of the in-stent lumen [17,57,58], making the performance of EID-CT in evaluating ISR limited/challenging, especially in stents of ≤3.0 mm [53,58,59]. PCD-CT can improve the evaluation of the in-stent lumen and minimize blooming artifacts lumen [17,58]. Few current studies in the literature have assessed the diagnostic performance of PCD-CT in the evaluation of stent patency compared to ICA as the reference standard. In this study, the meta-analysis of two studies [44,45] including 59 stents in 32 patients showed that UHR PCD-CT demonstrated a pooled sensitivity of 62.6% and specificity of 62.4% for detecting in-stent stenosis. A recent study by Qin et al. [37] evaluating 12 patients with 25 coronary stents who underwent both PCD-CT and ICA was evaluated. The authors reported that stent visualization was significantly improved with UHR PCD-CT combined with sharper reconstruction kernels (Bv72) resulting in higher diagnostic accuracy (88.0%).

Generally, assessing the small calcified crural vessels in the calf region using EID-CT angiography is more challenging due to difficulty in differentiation between true intravascular contrast and calcified plaques secondary to blooming artifacts [29,34,60,61]. The introduction of PCD-CT could improve the diagnosis of peripheral arterial stenosis, especially small calcified crural vessels, by improving the spatial resolution and reducing calcium-induced blooming artifacts [34,61]. A prospective study by Yalon et al. [61] compared lower extremity angiography using PCD-CTA and EID-CTA in 32 patients to evaluate infrapopliteal arterial stenoses. PCD-CTA demonstrated the superior visualization of small peripheral vessels, particularly fibular perforators, detected more clinically significant non-occlusive stenoses, and reduced calcium-related blooming artifacts that often appeared as total occlusions on EID-CTA.

It has been suggested that PCD-CT could improve the clinical decision-making in patients with coronary artery disease. The improved spatial resolution of PCD-CT reduces partial volume effects, enabling the more accurate visualization of luminal narrowing and more precise quantification of vascular stenosis [50,57,62]. PCD-CT has demonstrated excellent agreement with ICA for stenosis quantification compared with EID-CT [28,40,63]. Several studies have shown that PCD-CT indicates a lower percent diameter stenosis than EID-CT [28,40,64,65], especially for calcified plaques [40,66], leading to reclassification to a lower CAD-RADS category in 49–54% of patients [40,66]. A large study by Sakai et al. [46] including 7833 patients who underwent either PCD-CT (*n* = 3876) or EID-CT (*n* = 3957) showed that patients receiving PCD-CT had a lower referral rate to ICA than those undergoing EID-CT (9.9 vs. 13.1%) and that the rate of unnecessary diagnostic ICA was lower in the PCD-CT group than in the EID-CT cohort (5.6% vs. 8.4%). Among patients referred to subsequent ICA, the PCD-CT group had more frequent revascularization compared to the EID-CT group (43.3% vs. 35.5%). A recent study by Boussoussou et al. [38], using a decision and simulation model, showed that using PCD-CT compared to EID-CT reduced ICA referrals by 14.8%. As a result, PCD-CT can help avoid potential complications associated with ICA [50,67], especially in patients with advanced coronary atherosclerosis in whom invasive procedures are associated with an increased risk of embolic events [68].

Spectral imaging in PCD-CT allows post-processing reconstruction images in VMI, iodine maps, and VNCa images [39,41,58]. The selected energy level can affect image interpretation and diagnostic accuracy, so that the optimal energy level often depends on the clinical scenario [41,58,69]. A lower energy level, such as 40 keV, can improve image contrast, which can be used for an evaluation of subtle restenosis within stents [58,70]. In contrast, high energy levels, such as 100 keV, can reduce blooming artifacts associated with the stent or calcified plaques but compromise the iodine signal within the lumen [58]. Wolf et al. [41] investigated VMI levels (40–140 keV) for stenosis quantification in 33 patients with 64 stenoses, using QCA as a reference standard, and demonstrated that stenosis measurements were improved with higher VMI energy levels. However, the authors observed that the optimal VMI energy level differed according to the plaque composition. The lowest mean difference for diameter stenosis in the calcified plaque was 100 keV (17.2%); for mixed plaques, it was 140 keV (5%), and for non-calcified plaques, it was 40 keV (−0.5%). Rajiah et al. [69] confirmed that 100 keV VMI could improve the diagnostic confidence for an evaluation of stenosis associated with calcified plaque and stents.

The VNCa algorithm (PureLumen) is another spectral reconstruction for PCD-CT imaging [39]. VNCa algorithm subtracts calcifications from contrast-enhanced scans, minimizing the blooming artifacts associated with calcified plaques [39,57]. Our study indicates that the VNCa algorithm outperforms VMI in stenosis diameter measurements compared to 3D-QCA (mean difference: −10% vs. 0% for reader 1; mean difference: −7% vs. 1% for reader 2) [39]. Additionally, the VNCa algorithm outperformed conventional PCD-CT images in the detection of coronary stenosis with calcified plaques (median Agatston score of 1352) in terms of sensitivity (93% vs. 88%), specificity (80% vs. 69%), PPV (64% vs. 52%), NPV (96% vs. 94%) and accuracy (84% vs. 74%) [49]. The accurate stenosis measurements and detection via VNCa could be a result of the effective reduction of blooming artifacts associated with calcified plaques [39,57]. It has been suggested that evaluations of stenoses should be performed first on conventional PCD-CT images and then on VNCa images when blooming artifacts caused by calcified plaques are present [39]. This is because the evaluation of stenoses solely on VNCa images may lead to erroneous plaque subtraction, which may result in false diagnoses [39,71]. A conventional 320-MDCT scanner showed that calcium subtraction had a sensitivity of 89% and specificity of 85% in the detection of coronary stenosis with calcified plaques with a median Agatston score of 427 [72]. Importunately, calcium subtracted images in conventional EID-CT resulted from two different scans with different tube voltages, which may lead to misregistration artifacts and increased radiation doses [72,73]. On the other hand, VNCa images in PCD-CT can be generated from the original data by hiding the calcium components without additional radiation exposure [27,49].

Resolution modes and reconstruction kernels have affected PCD-CT performance [37,45,62]. The evaluation of luminal narrowing with UHR PCD-CT is more accurate than with SR and HR [30,37]. A study by Qin et al. [37], using ICA as the reference standard, reported that UHR PCD-CT had higher diagnostic accuracy than SR PCD-CT (88% vs. 78%) in coronary stent visualization. The UHR mode has higher spatial resolution, leading to fewer blooming artifacts and improved visualization of the lumen [37,45,57,62]. Sharp reconstruction kernels are preferable for assessing calcified plaques or in-stent stenoses because they can reduce calcium blooming and identify significant stenosis and IRS [37,45,62]. However, sharp kernels increase the image noise, which can be mitigated by increasing the slice thickness [62]. It has been demonstrated that combining UHR PCD-CT with a sharp kernel (Bv56, Bv60 and Bv72) results in the optimal results for stent visualization [37,45,74].

Beyond the improved diagnostic performance of PCD-CT, attributable to an enhanced spatial resolution and reduced blooming artifacts, the adoption of PCD-CT offers additional advantages [57,62]. PCD-CT enables high helical pitch acquisition (up to 3.2), allowing ultrafast scanning and an improved temporal resolution [57,69]. This is particularly beneficial for patients who are clinically unstable or unable to hold their breath [57], as well as for patients undergoing TAVR, who are challenging to scan in routine clinical practice due to frequent abnormal heart rhythms and contraindication to the use of beta-blockers to lower the heart rate or glycerol trinitrate for vasodilatation [43,44]. Another advantage of PCD-CT is the reduced volume of contrast media due to its inherent spectral reconstruction capabilities and shorter scan times [69,75,76].

It has been argued that PCD-CT may reduce the radiation dose compared to EID-CT. In our study, the mean effective radiation dose for PCD-CT ranged from 1.4 mSv to 20.5 mSv in the CAD evaluation. Two studies compared the mean radiation effective dose between EID-CT and PCD-CT in the evaluation of coronary stenoses. Vecsey-Nagy et al. [40] reported a mean effective radiation dose with UHR PCD-CT as lower than with EID-CT (14.9 vs. 12.3 mSv). On the other hand, Sakai et al. [46] reported that EID-CT had a lower mean effective radiation dose (9.0 mSv) than HR PCD-CT (11.6 mSv) or UHR PCD-CT (20.5 mSv). Variation across studies in the reported effective dose between EID-CT and PCD-CT could result from different resolution modes and image acquisition (prospective vs. retrospective ECG-gated) and scan parameters. Future studies employing more standardized image-acquisition protocols between the EID-CT and PCD-CT are needed to facilitate the reliable comparison of radiation doses.

This systematic review has several limitations that should be acknowledged. First, the number of studies included in the meta-analysis was small and primarily focused on coronary artery stenosis. Most studies were retrospective and single-center in design, which may limit the generalizability of the findings. Second, some studies specifically enrolled high-risk patients or those with severe calcifications; this may introduce selection bias and restrict the applicability of the results to the broader population. Third, the studies’ notable heterogeneity in imaging protocols, slice thicknesses, and reconstruction kernels might influence the pooled diagnostic estimates. As PCD-CT has been recently introduced in clinical practice and is still under development, the present findings reflect its current state of implementation and performance. Fourth, although invasive angiography served as the reference standard for vascular stenosis evaluation, methodological differences between modalities may contribute to measurement variability. Specifically, PCD-CT quantifies stenosis using a circular-equivalent diameter at the site of maximal narrowing, whereas invasive angiography estimates stenosis from 2D-dimensional-diameter reductions measured in orthogonal projections [30]. Finally, this review was restricted to studies published in English, which may introduce language and publication bias.

Future large prospective, multi-center studies with diverse patient populations are required to assess the utility of PCD-CT angiography in evaluating vascular stenoses in different territory vessels. Such studies should compare PCD-CT with advanced EID-CT systems, including 256- or 320-detector multidetector CT and dual-energy CT, using invasive angiography as the reference standard, and should assess not only diagnostic performance but also patient clinical outcomes to better evaluate the potential clinical benefits of PCD-CT. Furthermore, the standardization of PCD-CT acquisition and reconstruction protocols is needed to improve comparability across studies and to robustly validate diagnostic accuracy against invasive angiography.

## 5. Conclusions

This systematic review and meta-analysis demonstrates that PCD-CT angiography provides high diagnostic performance and improved stenosis quantification for coronary artery stenosis. UHR PCD-CT showed excellent accuracy for detecting coronary stenosis and consistently outperformed conventional EID-CT, especially in specificity and PPV. Evidence for the use of PCD-CT in evaluating stenosis in other vascular territories, including intracranial and peripheral arteries, remains limited but suggests promising diagnostic performance. PCD-CT represents a valuable advancement in vascular imaging, with further prospective multi-center studies needed to define its role across broader patient populations and vascular territories.

## Figures and Tables

**Table 1:** Characteristics and main findings of the included studies.

Author	Study Design/Sample Size	Mean Age (Years)	Vascular Territory/Vessel Status	Stenosis Detection Threshold	Reference Standard	CT Imaging Protocol	Mean Radiation Dose (mSv)	Main Findings
De Beukelaer, et al. [42]	Retrospective/18	59 ± 13	Intracranial/In-stent	≥50%	DSA	PCD-CT: ▪ PCD-CT scanner (NAEOTOM Alpha, Siemens Healthineers, Erlangen, Germany) ▪ UHR mode with Bv56 and Bv72 kernel ▪ Tube voltage 140 or 120 kvp and slice thickness of 0.2 mm	NR	- DSA identified relevant in-stent lumen narrowing in 18 of 162 vessel segments (11%). Also, DSA showed that 6 out of 18 patients (33%) had significant in-stent stenosis. - Based on segment analysis, PCD-CT had a sensitivity, specificity, PPV, and NPV of 100%, 89%, 53%, and 100%, respectively. - Interobserver agreement for diagnosing in-stent stenosis was excellent (κ = 0.92, *p* < 0.001).
Laux, et al. [36]	Retrospective/49	71 ± 9	Coronary/Native	≥50% and ≥70%	QCA	PCD-CT: ▪ PCD-CT scanner (NAEOTOM Alpha, Siemens Healthineers, Erlangen, Germany) ▪ UHR mode with Bv44 and Bv64 kernel ▪ Tube voltage 120 kVp; slice thicknesses of 0.6 mm (SR), 0.4 mm (HR), and 0.2 mm (UHR)	PCD-CT = 3.9	- Compared to the reference standard (QCA, 46.4%), the median percentage diameter stenosis measured via PCD-CT progressively decreased with an increasing spatial resolution: 61.4% with SR, 55.3% with HR, and 50.9% with UHR (all *p* < 0.001). - Mean difference between PCD-CT and QCA decreased from 13.2% (SR) to 5.2% (UHR). - LoA narrowed with higher resolution (SR: ±30.0%, HR: ±28.1%, UHR: ±23.0%). - A significant reduction in overestimation bias was observed only for calcified plaques as spatial resolution increased: bias declined from 15.8% (SR) to 11.0% (HR) and 4.3% (UHR), with progressively narrower LoA. - UHR reconstructions had superior diagnostic performance compared to SR and HR in detecting coronary stenosis ≥50% and ≥70%, as confirmed via QCA, at patient, vessel, and segment levels.
Mergen, et al. [39]	Retrospective/30	64 ± 8	Coronary/Native	Minimal to moderate coronary stenoses	QCA	PCD-CT: ▪ PCD-CT scanner (NAEOTOM Alpha, Siemens Healthineers, Erlangen, Germany) ▪ Qr44 kernel ▪ Tube voltage 120 kVp and 140 kVp, VMI, and VNCa images were reconstructed with slice thicknesses of 0.4 mm and 0.6 mm	NR	- Of 71 calcified stenoses, median diameter stenosis on 3D-QCA was 22% (IQR: 11–35%), while a median diameter stenosis on PCD-CT was on the following: ▪ Conventional VMI: 34% (IQR, 19–48%) for reader 1 and 32% (IQR, 15–45%) for reader 2. ▪ VNCa removal algorithm: 25% (IQR, 13–33%) for reader 1 and 19% (IQR, 12–30%) for reader 2. - VMI images overestimated stenosis compared to 3D-QCA: Reader 1: mean difference −10%, *p* < 0.001 Reader 2: mean difference −7%, *p* < 0.001 - VNCa images closely matched QCA in quantification stenosis: Reader 1: mean difference 0%, *p* = 0.68 Reader 2: mean difference 1%, *p* = 0.07 - VMI images led to an overestimation in 37% and 27% of calcified coronary stenoses; with VNCa images 8% and 7% of stenoses were overestimated.
Vecsey-Nagy, et al. [40]	Prospective/49	68.7 ± 9.2	Coronary/Native	≥10%	QCA	EID-CT: ▪ Dual-source CT (SOMATOM Force, Siemens Healthineers) ▪ 90–130 kVp and 150–600 mAs with Bv40 kernel ▪ 0.6 mm slice thickness PCD-CT: ▪ Dual-source PCD-CT (NAEOTOM Alpha, software version VA50, Siemens Healthineers) ▪ UHR acquisition mode with Bv64, Bv60, Bv56, Bv48 and Bv40 kernels. ▪ Tube voltage 120 kVp; slice thickness 0.2 mm	EID-CT = 14.9 PCD-CT = 12.3	- In 278 stenoses in 49 patients, the percentage diameter of stenosis measured via PCD-CT was significantly reduced compared to that measured via EID-CT, when using QCA as the reference standard, as follows: ▪ Calcified plaques (202/278): 45.1 ± 20.7% (PCD-CT) vs. 54.6 ± 19.2% (EID-CT), *p* < 0.001 ▪ Partially calcified plaques (51/278): 44.3 ± 19.6% (PCD-CT) vs. 54.9 ± 20.0% (EID-CT), *p* < 0.001 ▪ Non-calcified plaques (25/278): 39.1 ± 15.2% (EID-CT) vs. 39.0 ± 16.0% (EID-CT), *p* = 0.98 - In 12 patients (56 stenoses), UHR PCD-CT demonstrated higher agreement with QCA for stenosis diameter quantification (ICC: 0.91), with a mean difference of 7.3% and LoA from −10.7% to 25.2%, compared to EID-CT (ICC: 0.83), with a mean difference of 17.4% and LoA from −6.9% to 41.7%. - 49% of patients (24/49) were reclassified to a lower CAD-RADS category based on PCD-CT.
Wolf, et al. [41]	Retrospective/33	71.3 ± 9.0	Coronary/Native	≥20%	QCA	PCD-CT ▪ Dual-source PCD-CT scanner (NAEOTOM Alpha, Siemens Healthineers, Erlangen, Germany) ▪ VMI reconstructions were generated at energy levels of 40, 50, 60, 70, 80, 90, 100, 120, and 140 keV; slice thickness of 0.4 mm	PCD-CT = 2.0	- In 64 stenoses in 33 patients, PCD-CT scans were reconstructed at nine VMI energy levels from 40 to 140 keV. - The median percentage diameter of stenosis measured on PCD-CT progressively decreased with an increasing VMI level for calcified and mixed plaques: ▪ Calcified plaques (37/64, 57.8%): median percentage diameter stenosis decreased from 70.8% (40 keV) to 57.3% (140 keV); the lowest mean difference (17.2%) was at 100 keV. The corresponding median percentage diameter stenosis on QCA was 40.0 ± 8.7%. ▪ Mixed plaques (18/64, 28.1%): median percentage diameter stenosis decreased from 69.8% (40 keV) to 56.3% (140 keV); lowest mean difference (5.0%) was at 140 keV. The corresponding median percentage diameter stenosis on QCA was 51.3 ± 12.4%. - For non-calcified plaques (9/64, 14.1%): median percentage diameter stenosis was 46.6% at 40 keV vs. 54.6% at 140 keV; lowest mean difference (−0.5%) at 40 keV. The corresponding median percentage diameter stenosis on QCA was 47.1 ± 9.0%.
Soschynski, et al. [47]	Retrospective/92 (9 patients had ICA)	58 ± 14	Coronary/Native	≥50%	ICA	PCD-CT ▪ PCD-CT scanner (NAEOTOM Alpha, Siemens Healthineers, Erlangen, Germany) ▪ HR resolution mode with Bv40 kernel ▪ Tube voltage 120 kVp; slice thickness 0.4 mm	PCD-CT = 1.4	- Among 91 patients, 9 underwent both ICA and PCD-CT: ▪ Patient-level diagnostic performance of PCD-CTA: sensitivity 92%, specificity 96% ▪ Segment-level diagnostic performance: sensitivity 100%, specificity 67%
Augustin, et al. [34]	Retrospective/39	76.5 ± 12.2	Lower extremity arteries/Native	>60%	DSA	PCD-CT ▪ PCD-CT scanner (NAEOTOM Alpha, Siemens Healthineers, Erlangen, Germany) ▪ Standard resolution mode with Bv36, Bv48, and Bv56 kernels ▪ Tube voltage 140 kvp; slice thickness, 0.4 mm	NR	- In severely calcified crural vessels, sensitivity was consistent across all kernels (Bv36, Bv48, Bv56) at ~81%. - Specificity improved with increasing kernel sharpness: Bv36 (71.1%), Bv48 (76.9%), and Bv56 (79.6%). - The sharpest kernel (Bv56) reduced calcium blooming and improved specificity in severely calcified tibial vessels (Bv36 vs. Bv56, *p* = 0.024).
Fahrni, et al. [28]	Retrospective/26	64 ± 8	Coronary/Native	>50% and >70%	ICA	EID-CT: ▪ Dual-energy CT (Philips Healthcare, the Netherlands) ▪ 120 kVp and 255 mAs with XCB kernel ▪ 0.67 mm slice thickness PCD-CT: ▪ Single layer PCD-CT (Philips Healthcare, The Netherlands)—not yet FDA approved ▪ UHR mode with Detailed 2 kernel ▪ Tube voltage 120 kVp and slice thickness 0.25	NR	- In 26 patients (26 stenoses), PCD-CT demonstrated higher agreement with QCA (ICC: 0.94, *p* < 0.001; mean difference: 3 ± 7%; limits of agreement: ±13%, *p* < 0.001) compared to conventional EID-CT (ICC: 0.65, *p* = 0.006; mean difference: 0 ± 16%; limits of agreement: ±30%, *p* < 0.001). - PCD-CT demonstrated superior diagnostic performance in detecting coronary stenoses: ▪ For >50% diameter stenoses, PCD-CT had 100% sensitivity and 90% specificity, compared to 75% sensitivity and 50% specificity with EID-CT. ▪ For >70% stenoses, PCD-CT yielded 75% sensitivity and 100% specificity, compared to 37% and 83%, with EID-CT. - PCD-CT reclassified 38% (10/26) of stenoses into correct CAD-RADS categories that were misclassified via EID-CT.
Hagar, et al. [45]	Retrospective/18	83 ± 6	Coronary/In-stent	≥50%	ICA	PCD-CT: ▪ PCD-CT scanner (NAEOTOM Alpha, Siemens Healthineers, Erlangen, Germany) ▪ UHR mode with Bv-60 kernel ▪ Tube voltage 140 or 120 kVp; slice thickness of 0.2 mm	NR	- In 18 patients with 44 stents, UHR PCD-CTA achieved a diagnostic accuracy exceeding 88% for detecting in-stent stenosis of ≥ 50%, compared to the reference standard of ICA.
Kotronias, et al. [30]	Prospective/100	64	Coronary/Native	≥50%	QCA	PCD-CT: ▪ PCD-CT scanner (NAEOTOM Alpha, Siemens Healthineers, Erlangen, Germany) ▪ Bv40 Q4 and QR44 Q4 kernels for SR PCD-CT and Bv60 Q4 kernel for UHR PCD-CT ▪ Tube voltage 120 and 140 kVp; slice thickness were 0.4 mm (HR) and 0.2 mm (UHR)	PCD-CT: HR = 2.8 UHR = 7.5	- With 3D-QCA as reference standard, the percentage diameter stenosis measured via UHR PCD-CT was more accurate than HR PCD-CT (median difference: 3% vs. 6%; *p* < 0.001), with superior performance in severely calcified arteries (median difference: 3% vs. 6%; *p* = 0.002). - UHR PCD-CT showed a lower vessel-level CAD-RADS misclassification rate (7.6%) compared to HR PCD-CT (27.3%) when referenced to 3D QCA. - Per-vessel diagnostic accuracy was excellent for both HR PCD-CT (AUC: 0.94) and UHR PCD-CT (AUC: 0.99), with UHR-PCCTA showing significantly superior performance (ΔAUC: 0.05, *p* = 0.01).
Hagar, et al. [44]	Prospective/68	81 ± 7	Coronary/Native + In-stent	≥50% and ≥70%	ICA	PCD-CT: ▪ Dual-source PCD-CT scanner (NAEOTOM Alpha, Siemens Healthineers, Erlangen, Germany) ▪ UHR mode with Bv56 kernel ▪ Tube voltage 120 or 140 kVp; slice thickness were 0.2 mm	PCD-CT = 13.3	- In 68 patients, PCD-CT demonstrated a sensitivity of 96% and specificity of 84% for detecting ≥50% coronary diameter stenosis and sensitivity of 100% with specificity of 76% for detecting ≥70% stenosis. - In a subgroup of patients with severe calcification (24/68, 35%), PCD-CT maintained high diagnostic performance, showing 93% sensitivity and 70% specificity. - In a subgroup of patients with prior stent (15/68, 22%), PCD-CT showed 100% sensitivity and 86% specificity. - 54% (37 of 68) of patients could have avoided diagnostic ICA based on PCD-CT findings.
Brendel, et al. [43]	Retrospective/260	78.7 ± 8.1	Coronary/Native	≥50%	ICA	PCD-CT: ▪ PCD-CT scanner (NAEOTOM Alpha, Siemens Healthineers, Erlangen, Germany) ▪ HR mode with Qr40 kernel ▪ Tube voltage 120 kVp; slice thickness 0.4 mm	NA	- In 260 patients, PCD-CT demonstrated a sensitivity of 96% and specificity of 69% for detecting ≥50% coronary diameter stenosis.
Sakai, et al. [46]	Retrospective/7833 patients (3876 PCD-CT; 3957 EID-CT)	59.6 ± 11.6	Coronary/Native	≥50%	QCA	EID-CT: ▪ Revolution Apex 256 (GE HealthCare) and Aquilion ONE ViSION 320 (Canon Medical Systems) ▪ 100 to 120 kVp ▪ Slice thicknesses were 0.5 mm for Aquilion ONE ViSION 320 and 0.62 mm for Revolution Apex 256 PCD-CT: ▪ Dual-source PCD-CT (NAEOTOM Alpha, software version VA50, Siemens Healthineers) ▪ HR and UHR acquisition modes ▪ Tube voltage 140 kVp; slice thickness 0.4 mm (HR) and 0.2 mm (UHR)	-PCD-CT: HR = 11.6 UHR = 20.5 -EID-CT = 9.0	- Vessel-level analysis (*n* = 1686) showed that PCD-CT and EID-CT had comparable sensitivity (90.7% vs. 90.9%; *p* = 0.95), but PCD-CT demonstrated superior specificity (98.0% vs. 93.0%; *p* < 0.001) and accuracy (97.2% vs. 92.8%; *p* < 0.001). - Patients who underwent PCD-CT were less frequently referred to subsequent ICA than those undergoing EID-CT (9.9% vs. 13.1%; *p* < 0.001), with more revascularization if referred than EID-CT (43.4% vs. 35.5%).
Sharma, et al. [35]	Retrospective/60	79 ± 7	Coronary/Native	≥50%	QCA	PCD-CT: ▪ Dual-source PCD-CT scanner (NAEOTOM Alpha, Siemens Healthineers, Erlangen, Germany) ▪ Bv48 (HR); Bv48, Bv56, Bv64 (UHR); and Bv48, Bv56, Bv64 (adjusted UHR) ▪ Tube voltage 120 kVp (HR and UHR) and 90 kVP (adjusted UHR); slice thickness 0.4 mm (HR) and 0.2 mm (UHR and adjusted UHR)	NR	- Out of 60 patients, PCD-CT was performed using three scan modes: HR, UHR, and adjusted UHR, each in 20 patients. - In the per-patient analysis, both UHR and adjusted UHR modes achieved a sensitivity of 100% and a specificity of 60%; the HR mode demonstrated a lower sensitivity of 78% and specificity of 36%. - The area under the curve (AUC) did not differ significantly across modes: 57% for HR, and 80% for both UHR and adjusted UHR (*p* = 0.09).
Ghibes, et al. [29]	Retrospective/109	74.7 ± 11.1	Lower extremity arteries/Native	≥75%	DSA	PCD-CT: ▪ Spectral scanner PCD-CT scanner (NAEOTOM Alpha, Siemens Healthineers, Erlangen, Germany) ▪ HR mode ▪ Tube voltage 120 kVp; slice thickness 0.4 mm. ▪ Reconstructed at 70 keV, QIR3,1 mm; 55 keV, Bv36, 3 mm; and VNC (PureLumen; 70 keV, QIR3, 1 mm)	NR	- Per-segment analysis (*n* = 933): PCD-CT demonstrated high sensitivity (91%) and specificity (95%) for detecting high-grade vascular stenosis. - Pure lumen non-calcium reconstruction showed slightly reduced diagnostic performance (sensitivity: 85%, specificity: 89%) compared to standard PCD-CT.
Wang, et al. [48]	Prospective/122	~67 ± 10	Coronary/Native	≥50%	ICA	PCD-CT: ▪ Dual-source PCD-CT scanner (NAEOTOM Alpha, Siemens Healthineers, Erlangen, Germany) ▪ SR and UHR acquisition modes with Tube voltage 120 or 140 kVp: ○ SR mode reconstructed in normal (i.e., 70-keV VMI) and VNC (PureLumen) approaches with 0.6 mm and Bv40 kernel ○ UHR reconstructed in normal (i.e., 0.6-mm slice thickness, Bv40 kernel) and thin (i.e., 0.2-mm slice thickness, Bv64 kernel) approaches	PCD-CT SR = 7.4 UHR = 9.3	- PCD-CT UHRthin showed superior diagnostic performance vs. SRnormal, SRVNCa, and UHRnormal, with differences most pronounced at the segment level. - For vessels with an Agatston score >100, the diagnostic accuracy of SRVNCa (94.7%) and UHRthin (95.5%) was higher than that of SRnormal (88.6%) and UHRnormal (78.9%).
Qin, et al. [37]	Retrospective/69 (12 patients had ICA)	68.8 ± 8.9	Coronary/In-stent	≥50%	ICA	-PCD-CT: ▪ Dual-source PCD-CT scanner (NAEOTOM Alpha, Siemens Healthineers, Erlangen, Germany) ▪ Bv48 kernel for SR PCD-CT and Bv48, Bv56, Bv60, Bv64, Bv72, and Bv76 for UHR PCD-CT ▪ Tube voltage 120, and slice thickness were 0.6 mm (SR) and 0.2 mm (UHR)	PCD-CT = 8.5	- In 12 patients (25 stents), UHR-Bv72 PCD-CT demonstrated higher sensitivity (75% vs. 25%), specificity (90% vs. 89%), and diagnostic accuracy (88% vs. 78.3%) than SR PCD-CT for detecting significant in-stent stenosis, using ICA as the reference standard.
Nishihara, et al. [49]	Retrospective/17	76	Coronary/Native	≥50%	ICA	-PCD-CT: ▪ Dual-source PCD-CT scanner (NAEOTOM Alpha, Siemens Healthineers, Erlangen, Germany) ▪ HR mode with Bv44 kernel ▪ Tube voltage 140; slice thickness 0.4 mm	PCD-CT = 7.2	- In 17 patients (*n* = 162 segments) with median Agatston score was 1352, pure lumen non-calcium reconstruction showed higher diagnostic performance (sensitivity: 93%, specificity: 80%, PPV; 64%, NPV; 96%,) compared to standard PCD-CT images (sensitivity: 88%, specificity: 69% PPV; 52%, NPV; 94%) on-segment analysis level.
Boussoussou, et al. [38]	Retrospective/143 underwent PCD-CT and 109 underwent EID-CT	65 ± 9	Coronary/Native	≥50% and ≥70%	ICA	EID-CT: ▪ 256-slice (Philips iCT, Philips Healthcare) and a dedicated wide-detector cardiac CT scanner (GE CardioGraphe, GE Healthcare). ▪ 120 kVp ▪ Slice thicknesses were 0.62 mm for Philips iCT and 0.5 mm for GE CardioGraphe PCD-CT: ▪ Dual-source PCD-CT (NAEOTOM Alpha, software version VB10, Siemens Healthineers) ▪ Bv40 kernel with QIR 3 for SR PCD-CT and Bv56 with QIR 3 for UHR PCD-CT ▪ Tube voltage 120 and 140 kVp and slice thickness were 0.4 mm (HR) and 0.2 mm (UHR)	NR	- PCD-CT demonstrated higher diagnostic accuracy than EID-CT for both ≥50% and ≥70% coronary stenoses at all analysis levels: ▪ ≥50% stenosis: 88.1% vs. 77.9% (patient level), 91.6% vs. 77.8% (vessel level), 97.7% vs. 92.4% (segment level) ▪ ≥70% stenosis: 90.9% vs. 70.6% (patient level), 94.6% vs. 80.9% (vessel level), 98.6% vs. 94.1% (segment level) - PCD-CT demonstrated a 14.8% lower mean referral rate for ICA compared to EID-CT.
Demmert, et al. [50]	Retrospective/61	76 ± 9	Coronary/Native	>50% and >70%	ICA	PCD-CT: ▪ Dual source PCD-CT scanner (NAEOTOM Alpha, Siemens Healthineers, Erlangen, Germany) ▪ UHR mode with Bv60 kernel ▪ Tube voltage 120 or 140 kVp; slice thickness 0.2 mm	NR	- PCD-CT demonstrated high diagnostic performance across all Agatston score groups (600–999, 1000–1999, 2000–2999, >3000), maintaining excellent accuracy even at very high calcium scores (>3000), with sensitivity of 92–100% and specificity of 83–100% for detecting >50% stenosis and sensitivity of 88–100% and specificity of 89–100% for >70% stenosis.

DSA, digital subtraction angiography; EID-CT, energy-integrating detector-computed tomography; HR, high resolution; ICA, invasive coronary angiography; ICC, intraclass correlation coefficients; IQR, interquartile range; LoA, limits of agreement; mm, millimeter; NR, not reported; PCD-CT, photon-counting detector-computed tomography; QCA, quantitative coronary angiography; SR, standard resolution; TAVI, Transcatheter aortic valve implantation; UHR, ultra -high resolution; VMI, virtual monoenergetic images; VNC, virtual non-calcium.

**Table 2:** Quality assessment of included studies.

Study	Risk of Bias				Applicability Concerns		
	Patient Selection	Index Test	Reference Standard	Flow and Timing	Patient Selection	Index Test	Reference Standard
De Beukelaer, et al. [42]	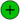	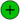	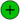	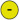	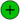	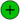	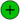
Laux, et al. [36]	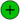	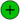	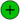	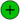	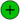	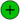	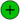
Mergen, et al. [39]	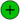	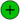	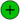	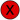	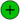	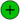	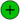
Vecsey-Nagy, et al. [40]	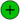	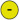	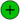	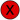	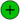	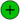	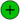
Wolf, et al. [41]	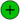	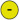	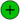	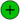	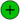	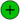	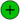
Soschynski, et al. [47]	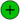	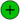	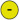	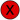	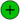	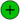	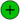
Augustin, et al. [34]	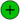	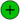	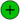	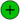	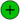	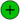	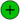
Fahrni, et al. [28]	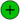	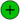	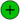	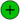	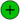	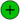	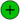
Hagar, et al. [45]	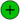	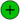	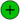	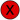	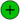	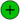	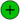
Kotronias, et al. [30]	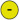	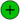	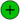	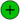	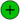	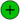	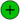
Hagar, et al. [44]	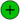	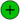	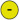	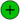	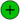	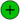	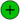
Brendel, et al. [43]	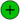	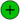	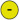	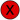	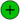	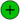	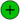
Sakai, et al. [46]	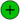	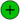	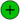	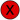	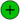	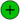	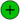
Sharma, et al. [35]	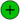	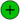	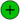	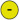	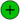	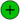	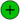
Ghibes, et al. [29]	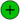	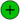	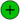	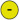	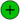	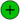	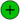
Wang, et al. [48]	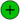	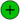	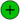	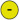	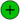	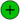	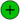
Qin, et al. [37]	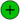	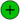	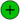	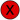	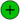	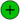	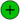
Nishihara, et al. [49]	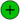	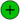	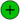	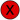	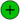	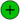	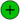
Boussoussou, et al. [38]	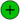	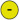	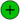	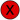	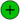	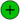	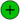
Demmert, et al. [50]	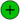	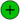	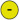	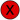	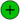	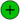	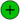

High risk (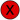), unclear risk (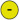), and low risk (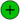).

**Table 3:** Pooled sensitivity, specificity, PPV, NPV, and AUC of PCD-CT angiography for detecting coronary artery stenosis in native and in-stents across different spatial resolution modes.

Spatial Resolution Mode	Vessel Status	Stenosis Detection Threshold	Analysis Level	Total Number of Patients, Vessel, or Segments/Number of Included Studies	Sensitivity % (95% CI)	Specificity % (95% CI)	PPV % (95% CI)	NPV % (95% CI)	AUC %
HR	Native	≥50%	Patient	269/2 studies	95.6 (90.3–98.1)	68.6 (60.4–75.7)	73.8 (66.6–79.9)	94.8 (88.0–97.6)	92.9
			Vessel	1540/2 studies	88.4 (84.3–91.6)	93.3 (55.6–99.4)	74.6 (52.6–88.6)	97.4 (86.7–99.5)	95.6
			Segment	288/2 studies	89.4 (78.2–95.1)	87.1 (42.7–98.4)	58.3 (40.3–74.3)	97.1 (84.9–99.5)	93.6
UHR	Native	≥50%	Patient	190/3 studies	96.1 (89.3–99.6)	87.5 (78.2–93.2)	91.9 (70.3–98.2)	94.8 (86.0–98.2)	96.7
			Vessel	632/3 studies	92.0 (81.5–97.7)	93.6 (90.2–95.8)	85.1 (65.4–94.5)	97.0 (94.0–98.6)	96.6
			Segment	1790/2 studies	95.8 (25.4–99.9)	97.5 (91.3–99.3)	76.3 (15.9–98.2)	99.6 (94.8–100)	98.2
		≥70%	Patient	129/2 studies	95.3 (85.1–99.6)	87.1 (50.5–97.8)	86.3 (22.5–99.3)	95.3 (78.6–99.1)	96.2
			Vessel	448/2 studies	92.5 (83.3–96.7)	93.3 (79.1–98.1)	70.9 (36.2–91.3)	98.6 (96.7–99.4)	96.7
	In-stent	≥50%	Patient	33/2 studies	62.6 (35.4–83.7)	62.4 (22.2–90.6)	68.6 (17.8–95.6)	57.2 (15–99.7)	65.8

AUC, area under curve; CI, confidence interval; EID-CT, energy-integrating detector-computed tomography; HR, high resolution; NPV, negative predictive value; PCD-CT, photon-counting detector-computed tomography; PPV, positive predictive value; UHR, ultra-high resolution.

## Data Availability

The data are contained within the article.

## Supplementary Materials

The following supporting information can be downloaded at: https://www.mdpi.com/article/10.3390/diagnostics16060881/s1, Table S1. Search strategy; Table S2. Studies excluded after full-text review and reasons for exclusion; Table S3. The reported diagnostic performance (sensitivity, specificity, positive predictive value, and negative predictive value) of PCD-CT angiography for stenosis detection in individual studies (*n* = 17); Table S4: The reported diagnostic performance (sensitivity, specificity, PPV, and NPV) of PCD-CT vs EID-CT angiography for detection of coronary artery stenosis; Figure S1. Forest plots of sensitivity (A) and specificity (B), and SROC curve (C) of high resolution PCDCT for detecting coronary ≥50% stenoses at the patient level; Figure S2. Forest plots of sensitivity (A) and specificity (B), and SROC curve (C) of high resolution PCDCT for detecting coronary ≥50% stenoses at the vessel level; Figure S3. Forest plots of sensitivity (A) and specificity (B), and SROC curve (C) of high resolution PCDCT for detecting coronary ≥50% stenoses at the segment level; Figure S4. Forest plots of sensitivity (A) and specificity (B), and SROC curve (C) of high resolution PCDCT for detecting coronary ≥50% stenoses at the patient level; Figure S5. Forest plots of sensitivity (A) and specificity (B), and SROC curve (C) of high resolution PCDCT for detecting coronary ≥50% stenoses at the vessel level; Figure S6. Forest plots of sensitivity (A) and specificity (B), and SROC curve (C) of high resolution PCDCT for detecting coronary ≥50% stenoses at the segment level; Figure S7. Forest plots of sensitivity (A) and specificity (B), and SROC curve (C) of high resolution PCDCT for detecting coronary ≥70% stenoses at the patient level; Figure S8. Forest plots of sensitivity (A) and specificity (B), and SROC curve (C) of high resolution PCDCT for detecting coronary ≥70% stenoses at the vessel level; Figure S9. Forest plots of sensitivity (A) and specificity (B), and SROC curve (C) of high resolution PCDCT for detecting coronary ≥50% in-stent restenosis at the patient level.
