# Supplementary material for: Diagnostic Performance of Photon-Counting CT Angiography in Vascular Stenosis Assessment: A Systematic Review and Meta-Analysis
Source: Diagnostics (Basel). 2026 Mar 16;16(6):881. doi: 10.3390/diagnostics16060881 (PMC13024787; doi:10.3390/diagnostics16060881)

Supplemental Material

Table S1. Search strategy

Databases	#	Searches
. Web of Science	1	TS=(("Vascular" OR "arterial" OR "artery" OR "arteries" OR "vessel" OR "blood vessel" OR "cardiovascular" OR "peripheral"))
	2	TS=(("stenosis" OR "occlusion" OR "plaque" OR "narrowing" OR "embolism" OR "stenotic" OR "thrombus" OR "thrombosis" OR "fibroatheroma" OR "blood clots" OR "constriction" OR "lumen reduction" OR "diminished diameter"))
	3	TS=(("Computed Tomography Angiography" OR "CT Angiography" OR "CTA" OR "CCTA" OR "CT Angio" OR "Angio-CT"))
	4	TS=(("photon*-count*" OR "spectra*" OR "multi-energ*"))
	5	#1 AND #2 AND #3 AND #4<br>Refined by: LANGUAGES: (ENGLISH) Timespan= Timespan: 1980-01-01 to 2025-12-30
PubMed	1	"Vascular"[All Fields] OR "arterial"[All Fields] OR "artery"[All Fields] OR "arteries"[All Fields] OR "vessel"[All Fields] OR "blood vessel"[All Fields] OR "cardiovascular"[All Fields] OR "peripheral"[All Fields]
	2	"stenosis"[All Fields] OR "occlusion"[All Fields] OR "plaque"[All Fields] OR "narrowing"[All Fields] OR "embolism"[All Fields] OR "stenotic"[All Fields] OR "thrombus"[All Fields] OR "thrombosis"[All Fields] OR "fibroatheroma"[All Fields] OR "blood clots"[All Fields] OR "constriction"[All Fields] OR "lumen reduction"[All Fields] OR "diminished diameter"[All Fields]
	3	"Computed Tomography Angiography"[All Fields] OR "CT Angiography"[All Fields] OR "CTA"[All Fields] OR "CCTA"[All Fields] OR "CT Angio"[All Fields] OR "Angio-CT"[All Fields]
	4	"photon* count*"[All Fields] OR "spectra*"[All Fields] OR "multi energ*"[All Fields]
	5	#1 AND #2 AND #3 AND #4<br>Filters: English, from 1980/1/1 - 2025/12/30
Cochrane	1	(("Vascular" OR "arterial" OR "artery" OR "arteries" OR "vessel" OR "blood vessel" OR "cardiovascular" OR "peripheral")):ti,ab,kw
	2	((("stenosis" OR "occlusion" OR "plaque" OR "narrowing" OR "embolism" OR "stenotic" OR "thrombus" OR "thrombosis" OR "fibroatheroma" OR "blood clots" OR "constriction" OR "lumen reduction" OR "diminished diameter"))):ti,ab,kw
	3	((("Computed Tomography Angiography" OR "CT Angiography" OR "CTA" OR "CCTA" OR "CT Angio" OR "Angio-CT"))):ti,ab,kw
	4	(((photon* NEXT count* OR spectra* OR multi-energ*))):ti,ab,kw
	5	#1 AND #2 AND #3 AND #4 with Cochrane Library publication date Between Jan 1980 and Dec 2025 and filtered in English

Table S2. Studies excluded after full-text review and reasons for exclusion

#	Study (Authors / Title)	Reason for exclusion
1	Ayx et al., 2025 / Feasibility of on-site CT-FFR analysis on cardiac photon-counting CT in evaluation of hemodynamically significant stenosis in comparison to invasive catheter angiography.	Not related to diagnostic accuracy or quantification (i.e., focused on functional assessment)
2	van der Bie et al.,2024 / Image quality assessment of coronary artery segments using ultra-high resolution dual source photon-counting detector computed tomography.	Focused on Image quality
3	Boriesosdick et al., 2025 / Deep Learning Based Detection of Large Vessel Occlusions in Acute Ischemic Stroke Using High-Resolution Photon Counting Computed Tomography and Conventional Multidetector Computed Tomography	No reference standard used
4	Brendel et al., 2025 / Coronary artery disease detection using deep learning and ultrahigh-resolution photon-counting coronary CT angiography.	No reference standard used
5	Chang et al., 2024 / Improving Stenosis Assessment in Energy Integrating Detector CT via Learned Monoenergetic Imaging Capability.	No reference standard used
6	De Beukelaer et al., 2025 / Photon-counting CT-angiography in comparison to digital subtraction angiography for assessing intracranial aneurysms after coiling or clipping	Did not assess vascular stenosis
7	Fahrni et al., 2025 / Ultra-high-resolution 40 keV virtual monoenergetic imaging using spectral photon-counting CT in high-risk patients for coronary stenoses.	No reference standard used
8	Halfmann et al., 2024 / Ultrahigh-Spatial-Resolution Photon-counting Detector CT Angiography of Coronary Artery Disease for Stenosis Assessment.	No reference standard used
9	He et al., 2025 / Increased diagnostic accuracy and better morphology characterization of unruptured intracranial aneurysm by ultra-high-resolution photon-counting detector CT angiography.	Did not assess vascular stenosis
10	Koos et al., 2025 / Learned high resolution energy-integrating detector CT angiography: Harnessing the power of ultra-high-resolution photon counting detector CT.	No reference standard used
11	Koons et al., 2024 / Learned high-resolution cardiac CT imaging from ultra-high-resolution PCD-CT.	No reference standard used
12	Koons et al., 2023 / Coronary artery stenosis quantification in patients with dense calcifications using ultra-high-resolution photon-counting-detector computed tomography.	No reference standard used
13	McCollough et al., 2023 / Comparison of Photon-counting Detector and Energy-integrating Detector CT for Visual Estimation of Coronary Percent Luminal Stenosis.	No reference standard used
14	Mergen et al., 2022 / First in-human quantitative plaque characterization with ultra-high resolution coronary photon-counting CT angiography.	Not related to diagnostic accuracy or quantification (i.e., focused on plaque characterisation)
15	Simon et al., 2024 / Photon-counting detector CT reduces the rate of referrals to invasive coronary angiography as compared to CT with whole heart coverage energy-integrating detector.	No reference standard used
16	Vecsey-Nagy et al., 2024 / Ultra-high resolution coronary CT angiography on photon-counting detector CT: bi-centre study on the impact of quantum iterative reconstruction on image quality and accuracy of stenosis measurements.	Focused on Image quality
17	Wolf et al., 2023 / Intra-individual comparison of coronary artery stenosis measurements between energy-integrating detector CT and photon-counting detector CT	No reference standard used
18	Yalon et al., 2024 / Infrapopliteal Segments on Lower Extremity CTA: Prospective Intraindividual Comparison of Energy-Integrating Detector CT and Photon-Counting Detector CT.	No reference standard used
19	Zsarnoczay et al., 2024 / Intra-individual comparison of coronary CT angiography-based FFR between energy-integrating and photon-counting detector CT systems	Not related to diagnostic accuracy or quantification (i.e., focused on functional assessment)
20	Tremamunno et al., 2025 / Semiquantitative metrics of coronary artery disease burden: Intra-individual comparison between ultrahigh-resolution photon-counting detector CT and energy-integrating detector CT.	No reference standard used

Table S3. The reported diagnostic performance (sensitivity, specificity, positive predictive value, and negative predictive value) of PCD-CT angiography for stenosis detection in individual studies (n=17)

Author	Vascular territory / Vessel status	Stenosis detection threshold	Analysis level	Total number of patients, vessel, or segments	PCD-CT Resolution	TP	FP	TN	FN	Sensitivity	Specificity	PPV	NPV
Laux, et al. [36]	Coronary / Native	≥50%	Patient	49	SR	NR	NR	NR	NR	100.0	29.6	53.7	100.0
			Patient	49	HR	NR	NR	NR	NR	95.5	33.3	53.9	90.0
			Patient	49	UHR	NR	NR	NR	NR	95.5	51.6	61.8	93.3
			Vessel	196	SR	NR	NR	NR	NR	97.0	68.7	38.6	99.1
			Vessel	196	HR	NR	NR	NR	NR	94.0	74.2	42.5	98.4
			Vessel	196	UHR	NR	NR	NR	NR	90.9	85.9	56.6	97.9
			Segment	874	SR	NR	NR	NR	NR	95.0	75.2	15.5	99.7
			Segment	874	HR	NR	NR	NR	NR	90.0	77.7	16.2	99.4
			Segment	874	UHR	NR	NR	NR	NR	82.5	83.1	19.0	99.0
		≥70%	Patient	49	SR	NR	NR	NR	NR	87.5	61.0	30.4	96.2
			Patient	49	HR	NR	NR	NR	NR	87.5	68.3	35.0	96.6
			Patient	49	UHR	NR	NR	NR	NR	100.0	80.5	50.0	100.0
			Vessel	196	SR	NR	NR	NR	NR	75.0	85.6	18.2	98.8
			Vessel	196	HR	NR	NR	NR	NR	75.0	87.8	20.7	98.8
			Vessel	196	UHR	NR	NR	NR	NR	100.0	94.2	42.1	100.0
			Segment	874	SR	NR	NR	NR	NR	63.6	84.2	4.9	99.5
			Segment	874	HR	NR	NR	NR	NR	63.6	85.5	5.3	99.5
			Segment	874	UHR	NR	NR	NR	NR	90.9	86.9	8.1	99.9
Soschynski, et al. [47]	Coronary / Native	≥50%	Patient	9	HR	3	2	4	0	100.0	67.0	60.0	100.0
			Segment	126	HR	12	5	108	1	92.0	96.0	71.0	99.0
Fahrni, et al. [28]	Coronary / Native	>50% (PCD-CT)	Segment	NR	UHR	NR	NR	NR	NR	100.0	90.0	94.0	100.0
		>70% (PCD-CT)	Segment	NR	UHR	NR	NR	NR	NR	75.0	100.0	10.00	90.0
		>50% (EID-CT)	Segment	NR	NA	NR	NR	NR	NR	75.0	50.0	71.0	56.0
		>70% (EID-CT)	Segment	NR	NA	NR	NR	NR	NR	37.0	83.0	50.0	75.0
Hagar, et al. [45]	Coronary / In-stent	≥50%	Patient	18	UHR	3*	4*	11*	0*	100.0 *	73.3 *	42.8 *	100.0 *
			Vessel	44	UHR	5*	4*	35*	0*	100.0 *	89.7 *	55.5 *	100.0 *
Kotronias, et al. [30]	Coronary / Native	≥50%	Vessel	139	HR	NA	NA	NA	NR	85.0	93.0	NR	NR
			Vessel	139	UHR	NR	NR	NR	NR	95.0	100.0	NR	NR
Hagar, et al. [44]	Coronary / native + In-stent	≥50%	Patient	68	UHR	23	7	37	1	96.0	84.0	77.0	97.0
			Vessel	204	UHR	31	15	154	4	89.0	91.0	67.0	97.0
			Segments	965	UHR	33	43	879	10	77.0	95.0	43.0	99.0
		≥70%	Patient	68	UHR	18	12	38	0	100.0	76.0	60.0	100.0
			Vessel	204	UHR	25	21	156	2	93.0	88.0	54.0	99.0
			Segments	965	UHR	23	53	883	6	79.0	94.0	30.0	99.0
	Coronary / In-stent	≥50%	Patient	15	UHR	8	1	0	6	100	86	89	100

Author	Vascular territory / Vessel status	Stenosis detection threshold	Analysis level	Total number of patients, vessel, or segments	PCD-CT Resolution	TP	FP	TN	FN	Sensitivity	Specificity	PPV	NPV
		≥70%	Patient	15	UHR	7	2	0	6	100	75	78	100
Brendel, et al. [43]	Coronary / Native	≥50%	Patient	260	HR	121	42	92	5	96.0	68.0	74.0	94.0
			Vessel	780	HR	200	108	444	28	89.0	80.0	65.0	94.0
Sakai, et al. [46]	Coronary / Native	≥50% (PCD-CT)	Vessel	760	HR	70	14	669	7	91.0	98.0	83.0	99.0
		≥50% (EID-CT)	Vessel	926	NA	97	57	762	10	91.0	93.0	63.0	99.0
Sharma, et al. [35]	Coronary / Native	≥50%	Patient	20	HR	NR	NR	NR	NR	78.0	36.0	50.0	67.0
			Patient	20	UHR	NR	NR	NR	NR	100.0	60.0	71.0	100.0
			Patient	20	Adjusted UHR	NR	NR	NR	NR	100.0	60.0	45.0	100.0
			Vessel	58	HR	NR	NR	NR	NR	83.0	63.0	37.0	94.0
			Vessel	58	UHR	NR	NR	NR	NR	67.0	72.0	38.0	89.0
			Vessel	56	Adjusted UHR	NR	NR	NR	NR	100.0	73.0	35.0	100.0
Wang, et al. [48]	Coronary / Native	≥50%	Patient	61	SR$_{normal}$	39.5*	2.5*	14.5*	4.5*	89.7*	85.3*	94.0*	76.4*
			Vessel	182	SR$_{normal}$	87*	11*	78*	6*	93.5*	87.6*	88.8*	92.9*
			Segments	788	SR$_{normal}$	143*	67.5*	566.5*	11*	92.9*	89.3*	67.9*	98.1*
			Patient	61	SR$_{VNC}$ (PureLumen)	39.5*	1*	16*	4.5*	89.7*	94.1*	92.1*	78.1*
			Vessel	182	SR$_{VNC}$ (PureLumen)	88*	6.5*	82.5*	5*	94.6*	92.6*	93.1*	94.3*
			Segments	788	SR$_{VNC}$ (PureLumen)	143.5*	51*	583*	10.5*	93.2*	91.9*	73.8*	98.2*
			Patient	61	UHR$_{normal}$	38.5*	6*	15*	1.5*	96.2*	71.4*	86.5*	90.8*
			Vessel	182	UHR$_{normal}$	66.5*	18*	96*	1.5*	97.8*	84.2*	87.7*	98.3*
			Segments	825	UHR$_{normal}$	119*	56*	645*	5*	96.0*	92.0*	68.0*	99.2*
			Patient	61	UHR$_{thin}$	40*	1*	20*	0*	100.0*	95.2*	97.6*	100.0*
			Vessel	182	UHR$_{thin}$	68*	5*	109*	0*	100.0*	95.6*	93.1*	100.0*
			Segments	825	UHR$_{thin}$	124*	9*	692*	0*	100.0*	98.7*	93.2*	100.0*
Qin, et al. [37]	Coronary / In-stent	≥50%	Vessel	25	SR	NR	NR	NR	NR	25.0	89.5	33.3	85.0
			Vessel	25	UHR	NR	NR	NR	NR	75.0	90.0	60.0	95.0
Nishihara, et al. [49]	Coronary / Native	≥50%	Segments	162	HR$_{VNC}$ (PureLumen)	41	23	95	3	93.2	80.5	64.1	96.9
			Segments	162	HR	39	36	82	5	88.6	69.5	52	94.3

Author	Vascular territory / Vessel status	Stenosis detection threshold	Analysis level	Total number of patients, vessel, or segments	PCD-CT Resolution	TP	FP	TN	FN	Sensitivity	Specificity	PPV	NPV
Boussoussou, et al. [38]	Coronary / Native	≥50% (PCD-CT)	Patient	143	Total PCD-CT (HR + UHR)	NR	NR	NR	NR	100	NA	88.1	NA
		≥50% (PCD-CT)	Vessel	572	Total PCD-CT (HR + UHR)	NR	NR	NR	NR	97.9	88.5	80.9	98.8
		≥50% (PCD-CT)	Segments	2431	Total PCD-CT (HR + UHR)	NR	NR	NR	NR	94.5	97.9	80.9	99.5
		≥50% (PCD-CT)	Patient	55	UHR	NR	NR	NR	NR	100	NA	90.9	NA
		≥50% (PCD-CT)	Vessel	NR	UHR	NR	NR	NR	NR	100	87.1	81.8	100.0
		≥50% (PCD-CT)	Segments	NR	UHR	NR	NR	NR	NR	98.7	98.6	86.4	99.8
		≥70% (PCD-CT)	Patient	143	Total PCD-CT (HR + UHR)	NR	NR	NR	NR	98.9	72.7	89.1	96.9
		≥70% (PCD-CT)	Vessel	572	Total PCD-CT (HR + UHR)	NR	NR	NR	NR	94.5	94.6	85.6	98.1
		≥70% (PCD-CT)	Segments	2431	Total PCD-CT (HR + UHR)	NR	NR	NR	NR	93.8	98.9	85.8	99.6
		≥70% (PCD-CT)	Patient	55	UHR	NR	NR	NR	NR	96.9	86.9	91.2	95.2
		≥70% (PCD-CT)	Vessel	NA	UHR	NR	NR	NR	NR	94.1	96.5	88.9	98.2
		≥70% (PCD-CT)	Segments	NA	UHR	NR	NR	NR	NR	96.2	99.1	86.2	99.8
		≥50% (EID-CT)	Patient	109	NA	NR	NR	NR	NR	100.0	NA	77.8	NA
		≥50% (EID-CT)	Vessel	436	NA	NR	NR	NR	NR	93.5	71.6	56.4	96.6
		≥50% (EID-CT)	Segments	1853	NA	NR	NR	NR	NR	80.0	93.5	50.0	98.0
		≥70% (EID-CT)	Patient	109	NA	NR	NR	NR	NR	90.0	28.6	72.8	58.8
		≥70% (EID-CT)	Vessel	436	NA	NR	NR	NR	NR	79.0	81.5	56.0	92.8
		≥70% (EID-CT)	Segments	1853	NA	NR	NR	NR	NR	65.7	95.8	49.3	97.8
Demmert, et al. [50]	Coronary / Native	>50%	Patient	61	UHR	37*	2*	20*	2*	94.87*	90.91*	94.87*	90.91*
		>50%	Vessel	244	UHR	66*	9*	162*	7*	90.41*	94.74*	88*	95.86*
		>70%	Patient	61	UHR	35*	1*	23*	2*	94.59*	95.83*	97.22*	92.00*

Author	Vascular territory / Vessel status	Stenosis detection threshold	Analysis level	Total number of patients, vessel, or segments	PCD-CT Resolution	TP	FP	TN	FN	Sensitivity	Specificity	PPV	NPV
		>70%	Vessel	244	UHR	37*	7*	197*	3*	92.50*	96.57*	84.09*	98.50*
Augustin, et al. [34]	Lower extremity / Native	>60%	Segment	824	SR (Bv36 Kernal)	NR	NR	NR	NR	81.5	71.1	NR	NR
		>60%	Segment	824	SR (Bv48 Kernal)	NR	NR	NR	NR	81.5	76.8	NR	NR
		>60%	Segment	824	SR (Bv56 Kernal)	NR	NR	NR	NR	80.9	79.6	NR	NR
Ghibes, et al. [29]	Lower extremity / Native	≥75%	Segments	933	HR	NR	NR	NR	NR	91.0	95.0	91.0	94.0
		≥75%	Segments	780	HR_{VNC} (PureLumen)	NR	NR	NR	NR	85.0	89.0	83.0	90.0
De Beukelaer, et al. [42]	Intracranial / In-stent	≥50%	Segment	162	UHR	18	16	128	0	100.0	89.0	53.0	100.0

* Average across 2 readers

EID-CT energy-integrating detector- computed tomography, FN false-negative, FP false-positive , HR high resolution, NA not applicable, NR not reported, NPV negitive predictive value ,PCD-CT photon-counting detector- computed tomography, PPV positive predictive value, SR standard resolution, TN true-negative, TP true-positive, UHR ultra hight resolution, VNC virtual non-calcium

Table S4: The reported diagnostic performance (sensitivity, specificity, PPV, and NPV) of PCD-CT vs EID-CT angiography for detection of coronary artery stenosis

Vascular territory / Vessel status	Stenosis detection threshold	Analysis level	CT Imaging	Diagnostic performance	Number of studies / references
Coronary	≥50%	Patient	PCD-CT	Sensitivity= 100.0<br>Specificity= NA<br>PPV= 88.1<br>NPV= NA	1 / Boussoussou, et al. [38]
			EID-CT	Sensitivity= 100.0<br>Specificity= NA<br>PPV= 77.8<br>NPV= NA	
		Vessel	PCD-CT	Sensitivity=91.0–97.9<br>Specificity=88.5–98<br>PPV=80.9–93.0<br>NPV=99.0 –99.8	2 / Sakai et al., 2025 and Boussoussou, et al. [38]
			EID-CT	Sensitivity=91.0–93.5<br>Specificity=71.6–93.0<br>PPV=56.4–63.0<br>NPV=96.6–99.0	
		Segments	PCD-CT	Sensitivity=94.5–100<br>Specificity=90–97.9<br>PPV=80.9–94.0<br>NPV=99.5–100.0	2 / Fahrni, et al. [28] and Boussoussou, et al. [38]
			EID-CT	Sensitivity=75.0–80.0<br>Specificity=50.0–93.5<br>PPV=50.0–71.0<br>NPV=56.0–98.0	
	≥70%	Patient	PCD-CT	Sensitivity= 99.9<br>Specificity= 72.7<br>PPV= 89.1<br>NPV= 96.9	1 / Boussoussou, et al. [38]
			EID-CT	Sensitivity= 90.0<br>Specificity= 28.8<br>PPV= 72.8<br>NPV= 58.8	

		Vessel	PCD-CT	Sensitivity= 94.5<br>Specificity= 94.5<br>PPV= 85.0<br>NPV= 98.1	1 / Boussoussou, et al. [38]
			EID-CT	Sensitivity= 79.0<br>Specificity= 81.5<br>PPV= 56.0<br>NPV= 92.8	
		Segments	PCD-CT	Sensitivity=75.0–93.8<br>Specificity=98.9–100.0<br>PPV=85.8–100.0<br>NPV=90.0–99.6	2 / Fahrni, et al. [28] and Boussoussou, et al. [38]
			EID-CT	Sensitivity=37–65.7<br>Specificity=83–95.8<br>PPV=49.3–50.0<br>NPV=75.0–97.8	

EID-CT energy-integrating detector- computed tomography, NA not applicable, NPV negative predictive value, PCD-CT photon-counting detector- computed tomography, PPV positive predictive value,

Figure S1. Forest plots of sensitivity (A) and specificity (B), and SROC curve (C) of high resolution PCD-CT for detecting coronary ≥50% stenoses at the patient level

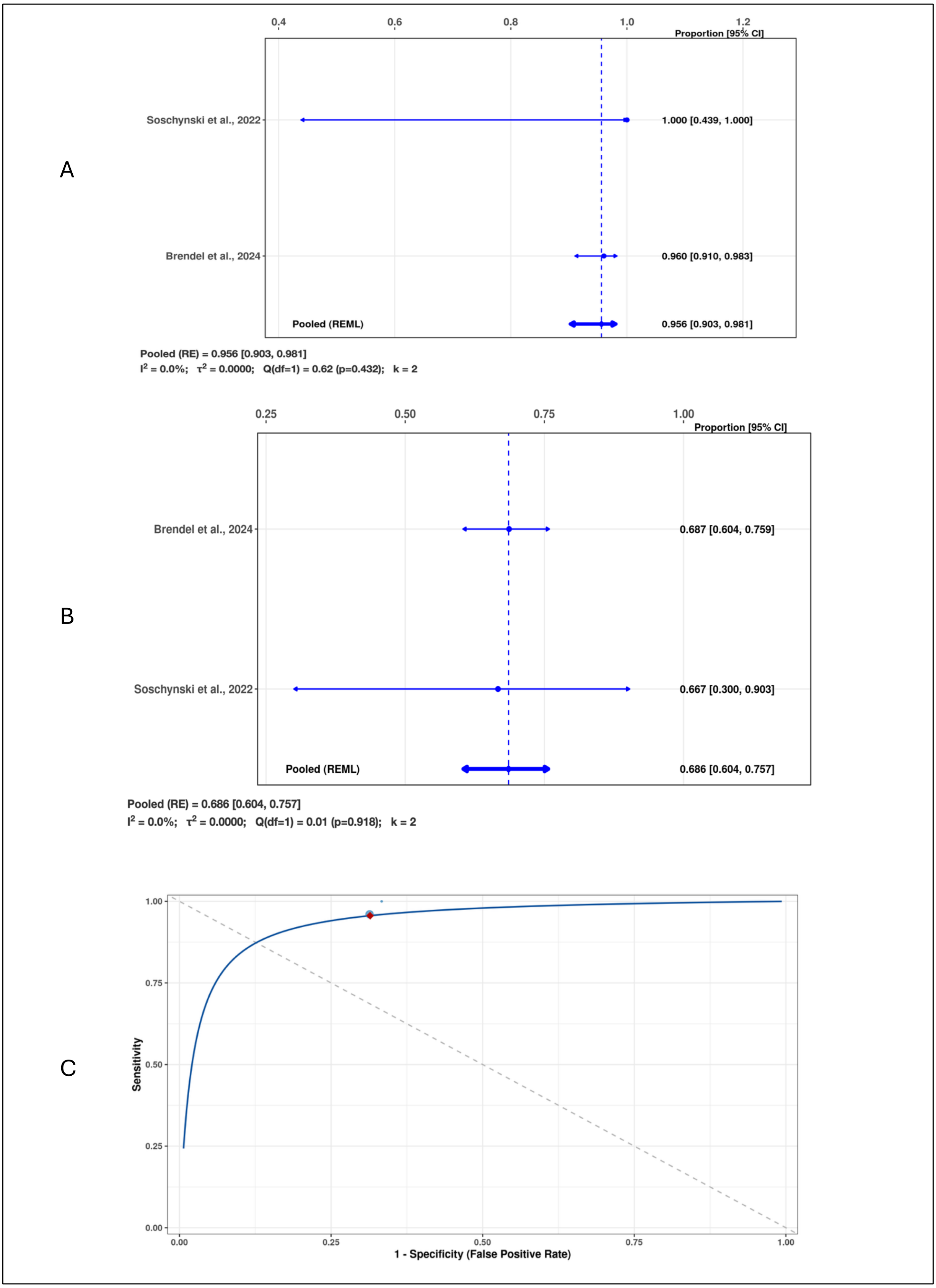

Figure S2. Forest plots of sensitivity (A) and specificity (B), and SROC curve (C) of high resolution PCD-CT for detecting coronary ≥50% stenoses at the vessel level

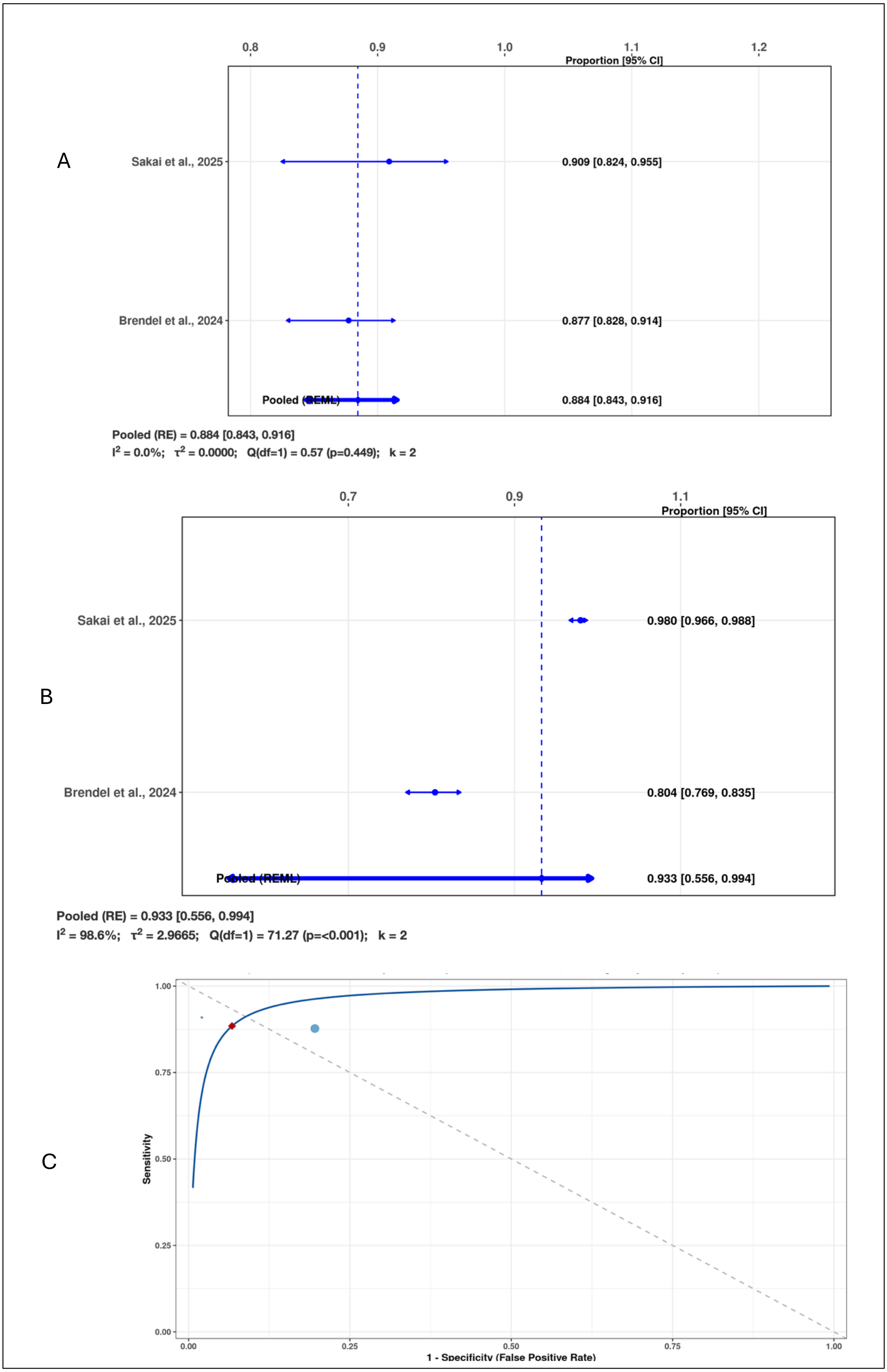

Figure S3. Forest plots of sensitivity (A) and specificity (B), and SROC curve (C) of high resolution PCD-CT for detecting coronary ≥50% stenoses at the segment level

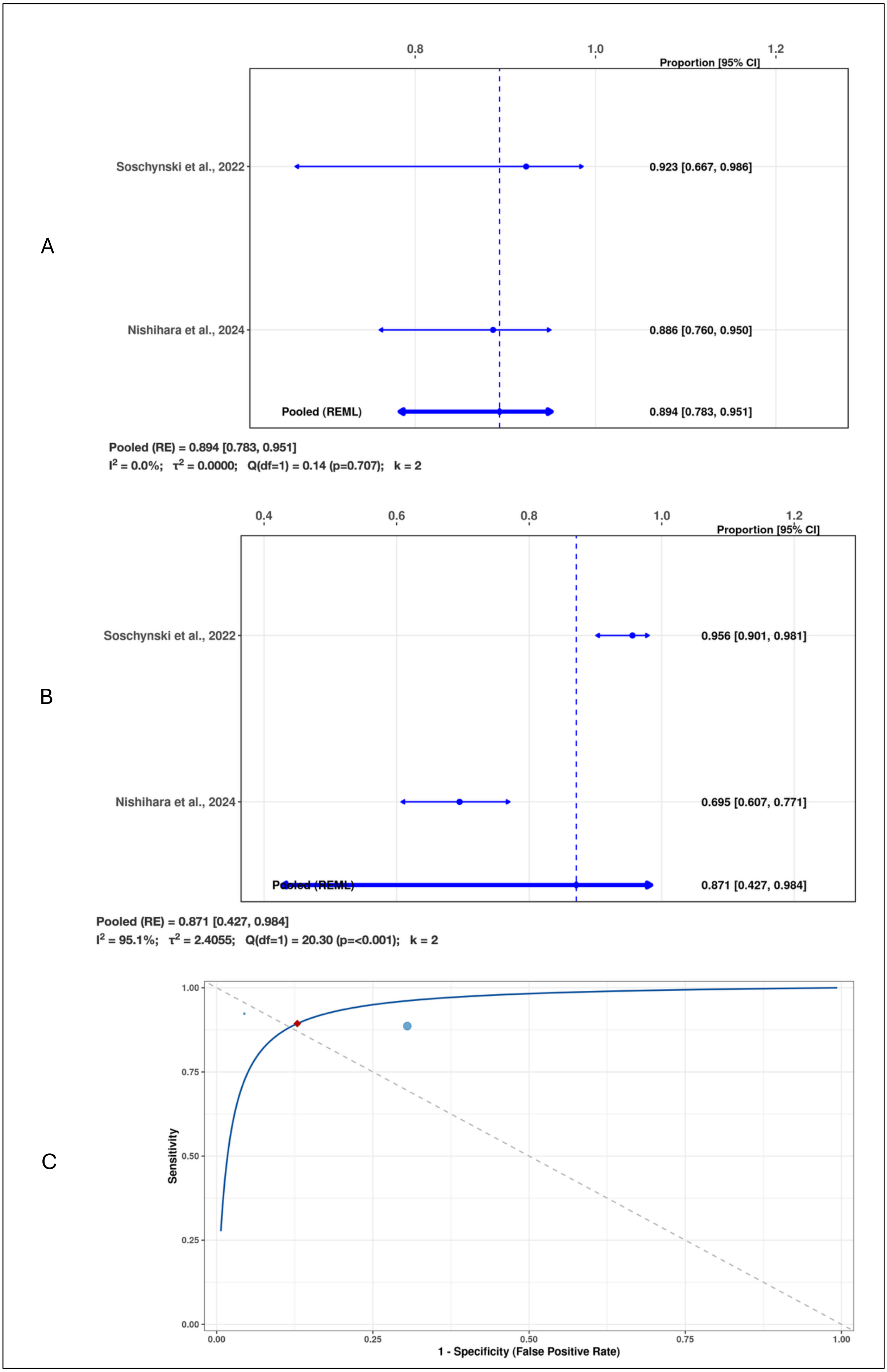

Figure S4. Forest plots of sensitivity (A) and specificity (B), and SROC curve (C) of high resolution PCD-CT for detecting coronary ≥50% stenoses at the patient level

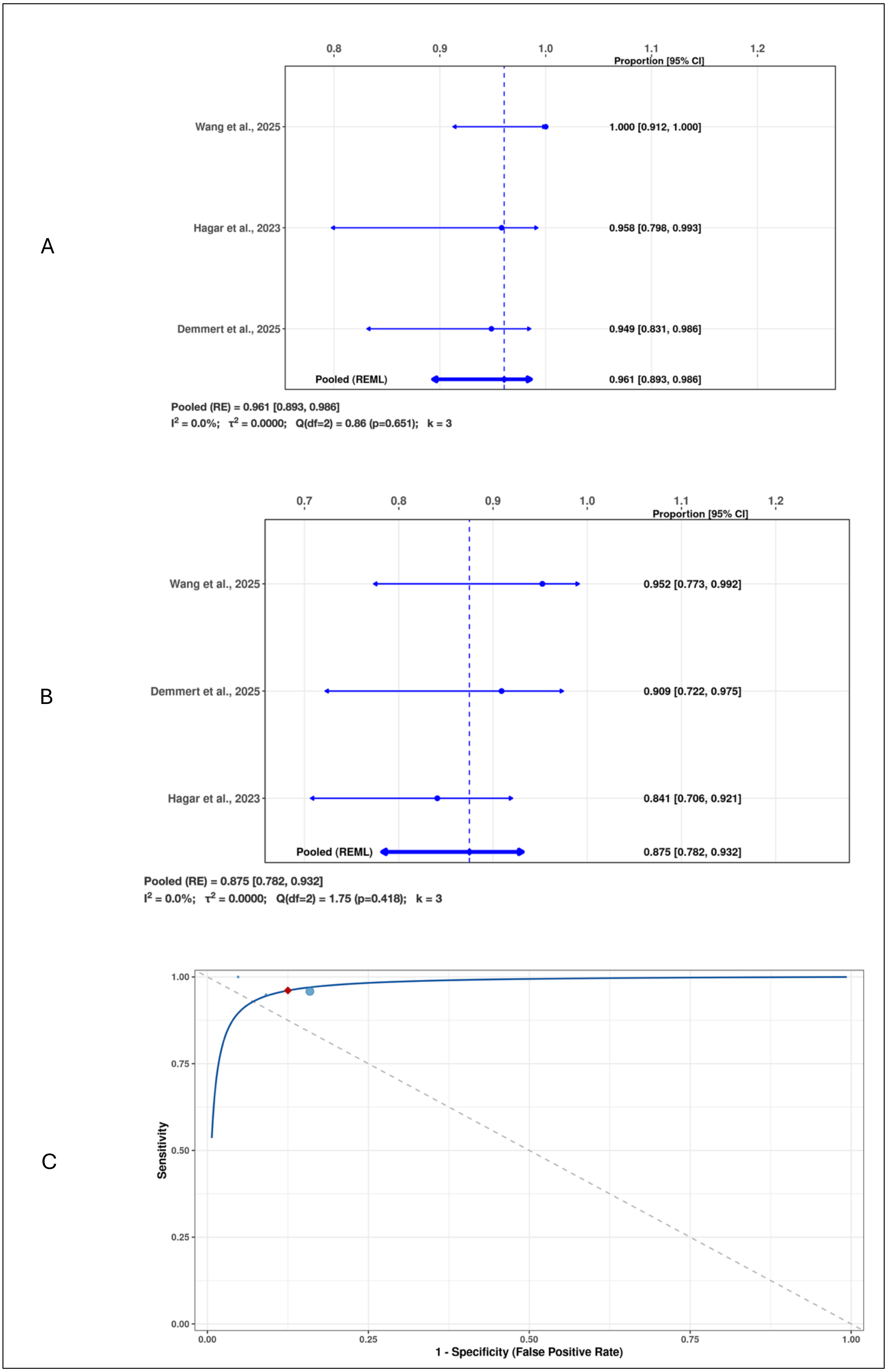

Figure S5. Forest plots of sensitivity (A) and specificity (B), and SROC curve (C) of high resolution PCD-CT for detecting coronary ≥50% stenoses at the vessel level

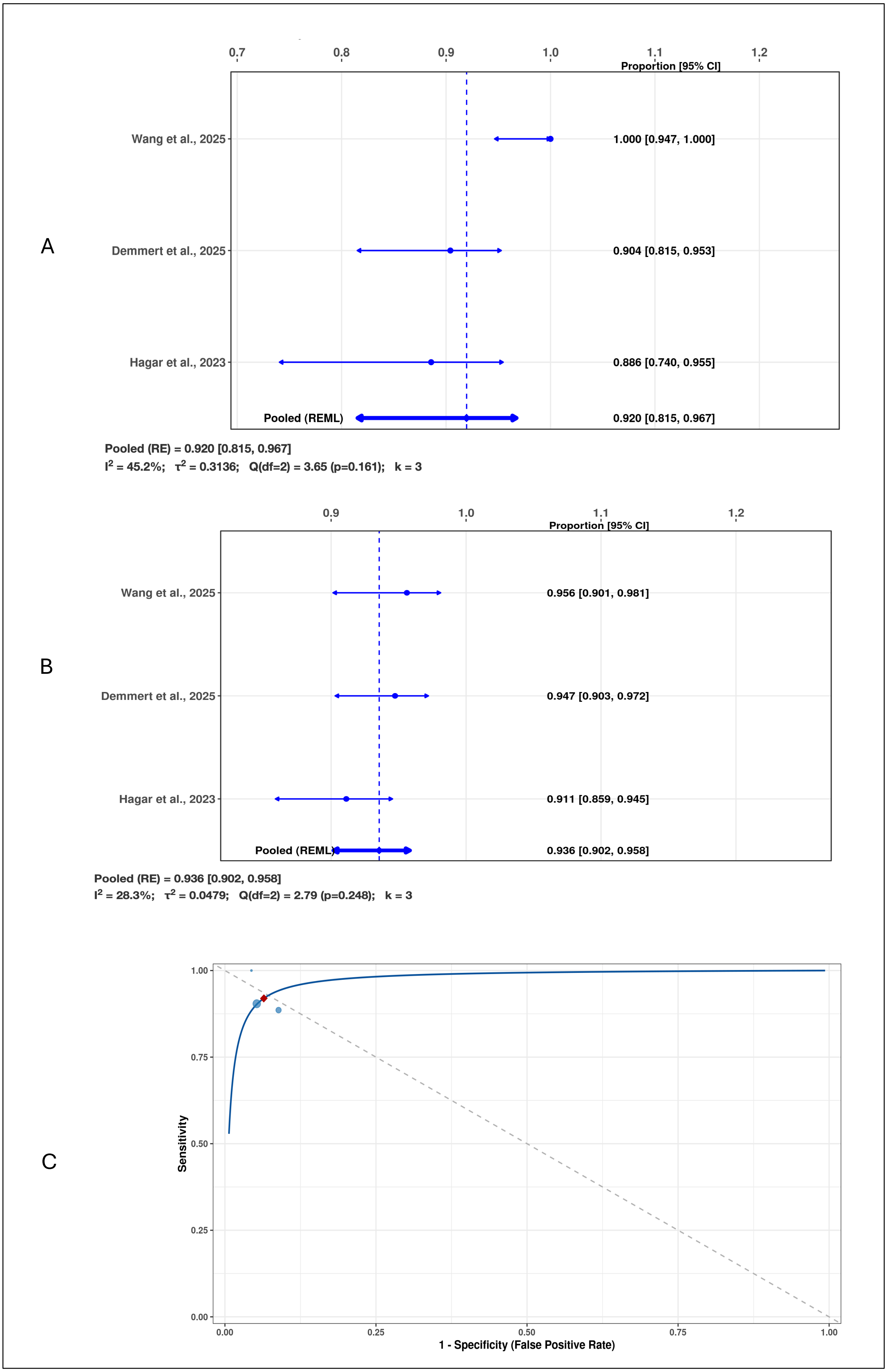

Figure S6. Forest plots of sensitivity (A) and specificity (B), and SROC curve (C) of high resolution PCD-CT for detecting coronary ≥50% stenoses at the segment level

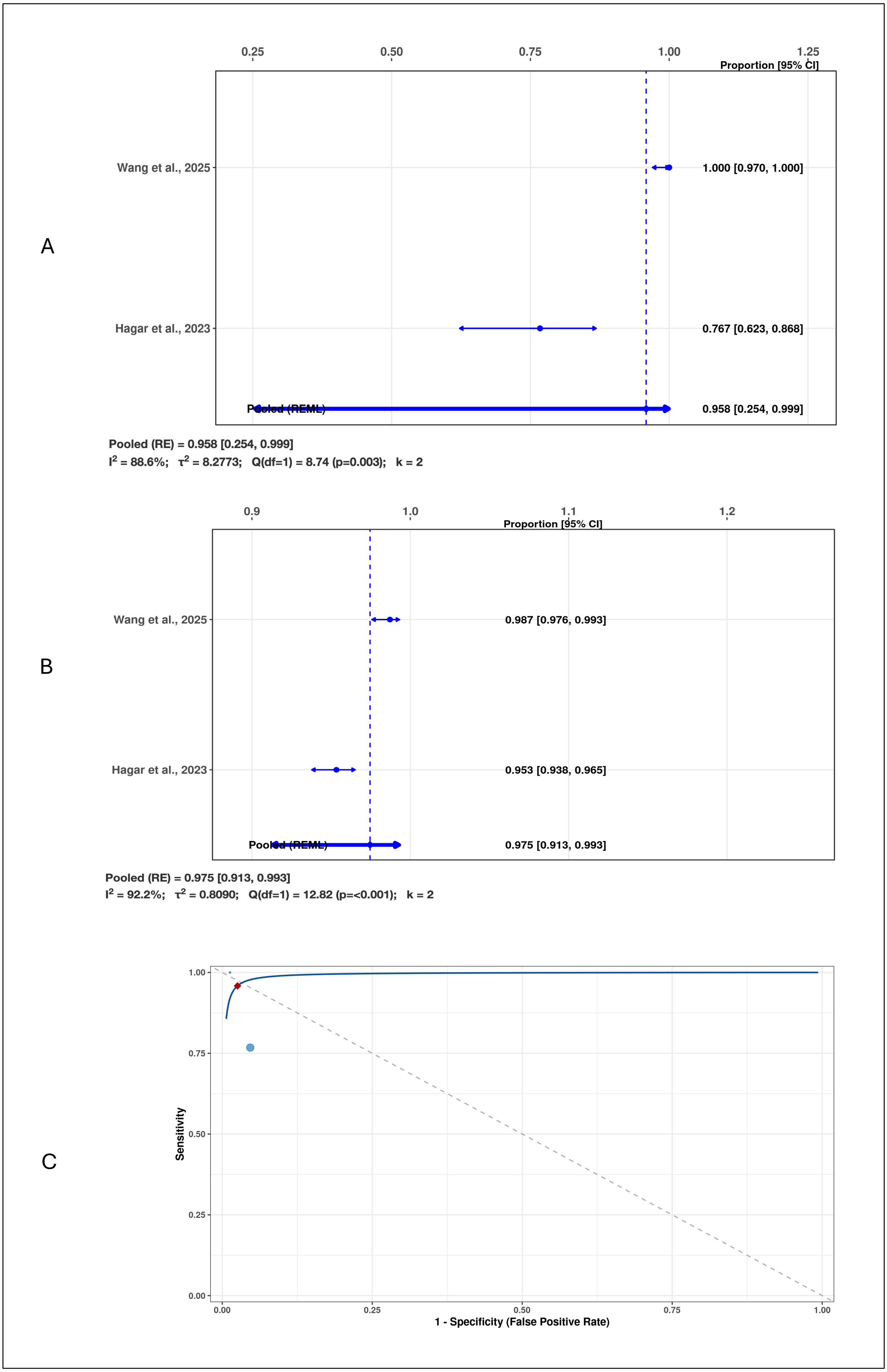

Figure S7. Forest plots of sensitivity (A) and specificity (B), and SROC curve (C) of high resolution PCD-CT for detecting coronary ≥70% stenoses at the patient level

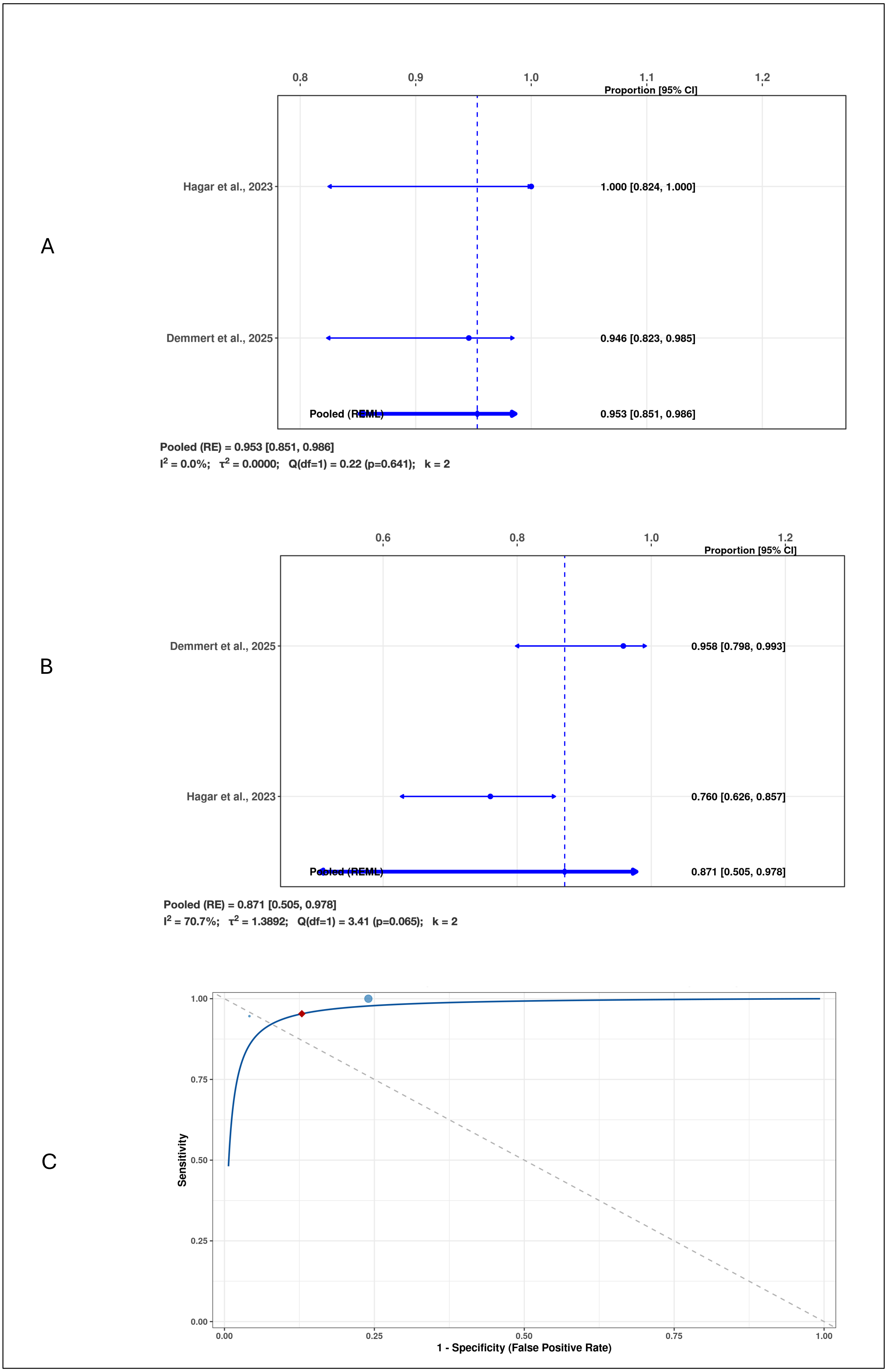

Figure S8. Forest plots of sensitivity (A) and specificity (B), and SROC curve (C) of high resolution PCD-CT for detecting coronary ≥70% stenoses at the vessel level

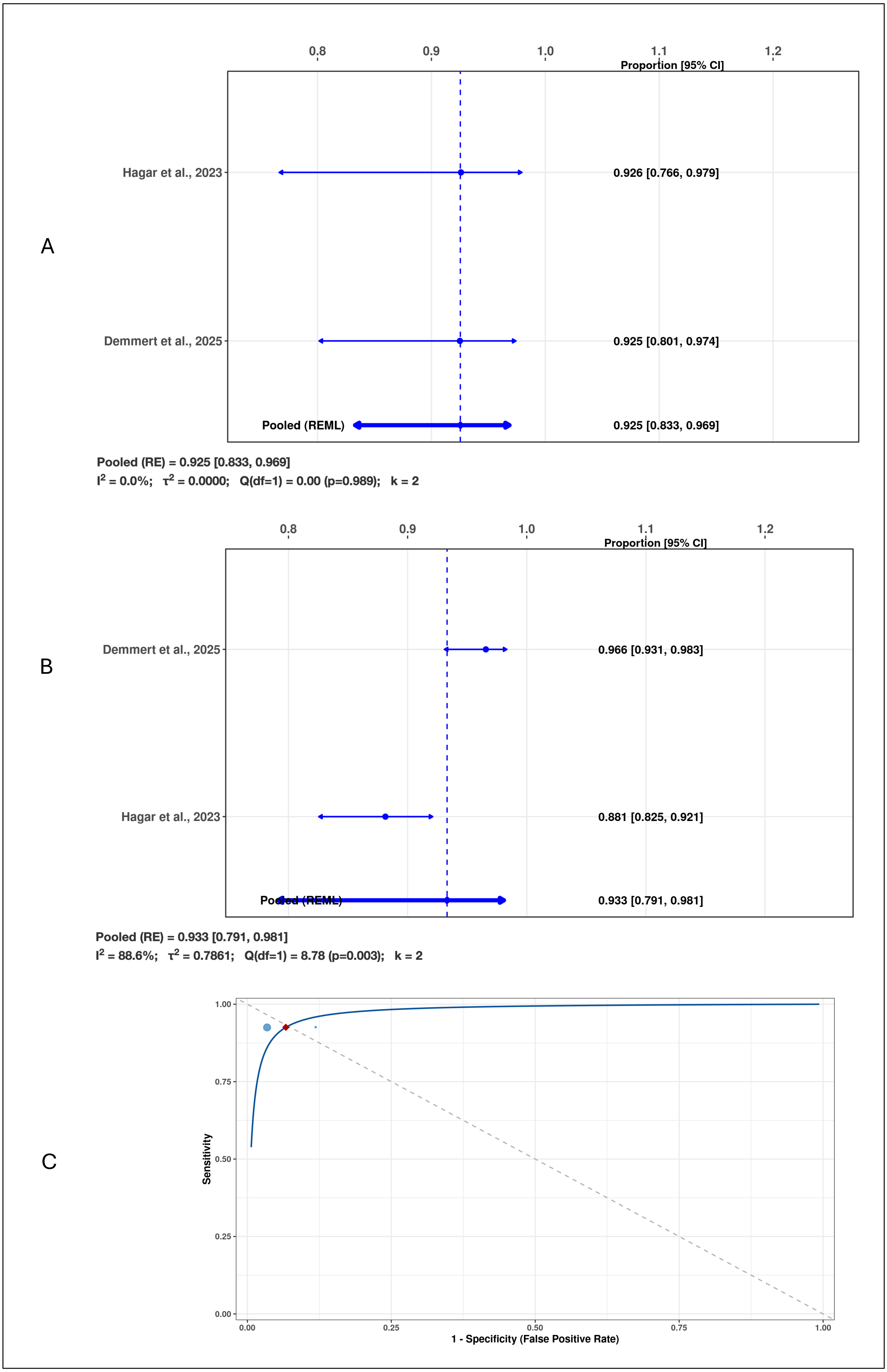

Figure S9. Forest plots of sensitivity (A) and specificity (B), and SROC curve (C) of high resolution PCD-CT for detecting coronary ≥50% in-stent restenosis at the patient level

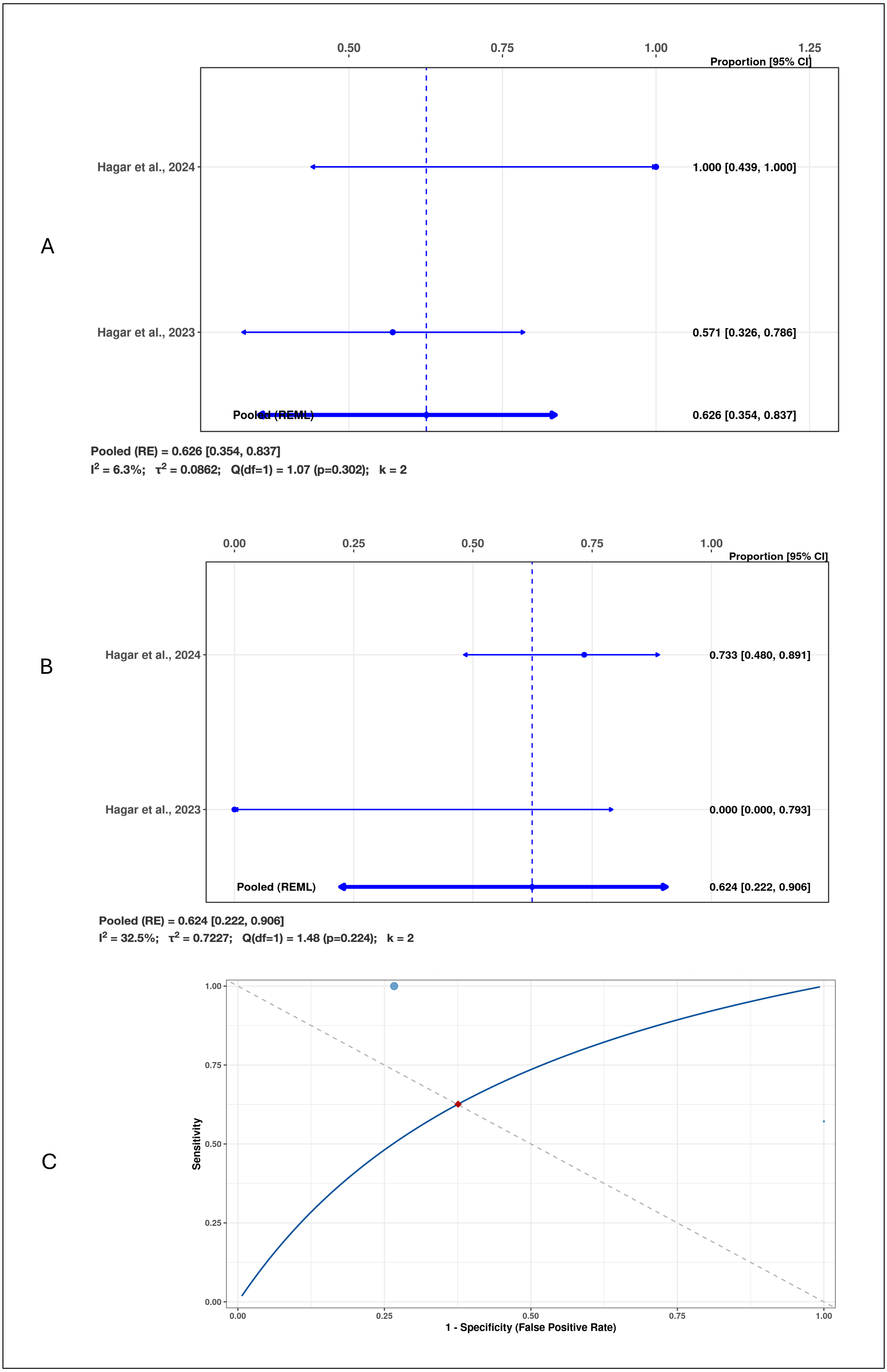

Supplement: Supplementary file 1 [file diagnostics-16-00881-s001.zip › diagnostics-4187792-supplementary resubmitted.pdf]
